# New therizinosaurid dinosaur from the marine Osoushinai Formation (Upper Cretaceous, Japan) provides insight for function and evolution of therizinosaur claws

**DOI:** 10.1038/s41598-022-11063-5

**Published:** 2022-05-03

**Authors:** Yoshitsugu Kobayashi, Ryuji Takasaki, Anthony R. Fiorillo, Tsogtbaatar Chinzorig, Yoshinori Hikida

**Affiliations:** 1 Hokkaido University Museum, Hokkaido University, Sapporo, Hokkaido 060-0810 Japan; 2 Faculty of Biosphere-Geosphere Science, Okayama University of Science, Okayama, 700-0005 Japan; 3 Huffington Department of Earth Sciences, Southern Methodist University, Dallas, TX 75275 USA; 4 Department of Biological Sciences, North Carolina State University, Raleigh, NC 27695 USA; 5 Division of Vertebrate Paleontology, Institute of Paleontology, Mongolian Academy of Sciences, Ulaanbaatar, 15160 Mongolia; 6 Nakagawa Museum of Natural History, Nakagawa, Hokkaido 098-2626 Japan

**Keywords:** Palaeontology, Speciation, Taxonomy

## Abstract

The record of therizinosaurs is rich in Asian countries such as Mongolia and China. Fragmentary therizinosaur specimens have been reported from the Lower and Upper Cretaceous deposits in Japan. One of these specimens, from the lower Campanian Osoushinai Formation in Nakagawa Town of Hokkaido Prefecture, was previously identified as a maniraptoran theropod dinosaur, possibly therizinosaur, but its taxonomic status remained unresolved. This study re-examines the specimen and provides a more detailed description and attempts to resolve its taxonomic status. Our study demonstrates that it is a new taxon, *Paralitherizinosaurus japonicus* gen. et sp. nov., because it shows a unique combination of characters in the metacarpal I and unguals. Our phylogenetic analysis places this new taxon within an unresolved clade of Therizinosauridae in the strict consensus tree. The 50% majority-rule consensus tree shows better resolution within Therizinosauridae, showing an unresolved monophyletic clade of *Paralitherizinosaurus, Therizinosaurus*, *Suzhousaurus*, and the Bissekty form. Geometric morphometric analysis suggests that *Paralitherizinosaurus* unguals most closely resemble *Therizinosaurus* unguals in being slender and has weak flexor tubercles. This study also shows an evolutionary trend in ungual shape, which associates a decrease in mechanical advantage, development of flexor tubercle, and hypothesized output (product of mechanical advantage and development of flexor tubercle) in derived therizinosaurs, supporting the hook-and-pull function of claws to bring vegetation to its mouth. *Paralitherizinosaurus* is the youngest therizinosaur from Japan and the first recovered from the marine deposits in Asia. This suggests a long temporal existence of therizinosaurs at the eastern edge of the Asian continent and adaptation of therizinosaurs to coastal environments.

## Introduction

**Figure 1:**
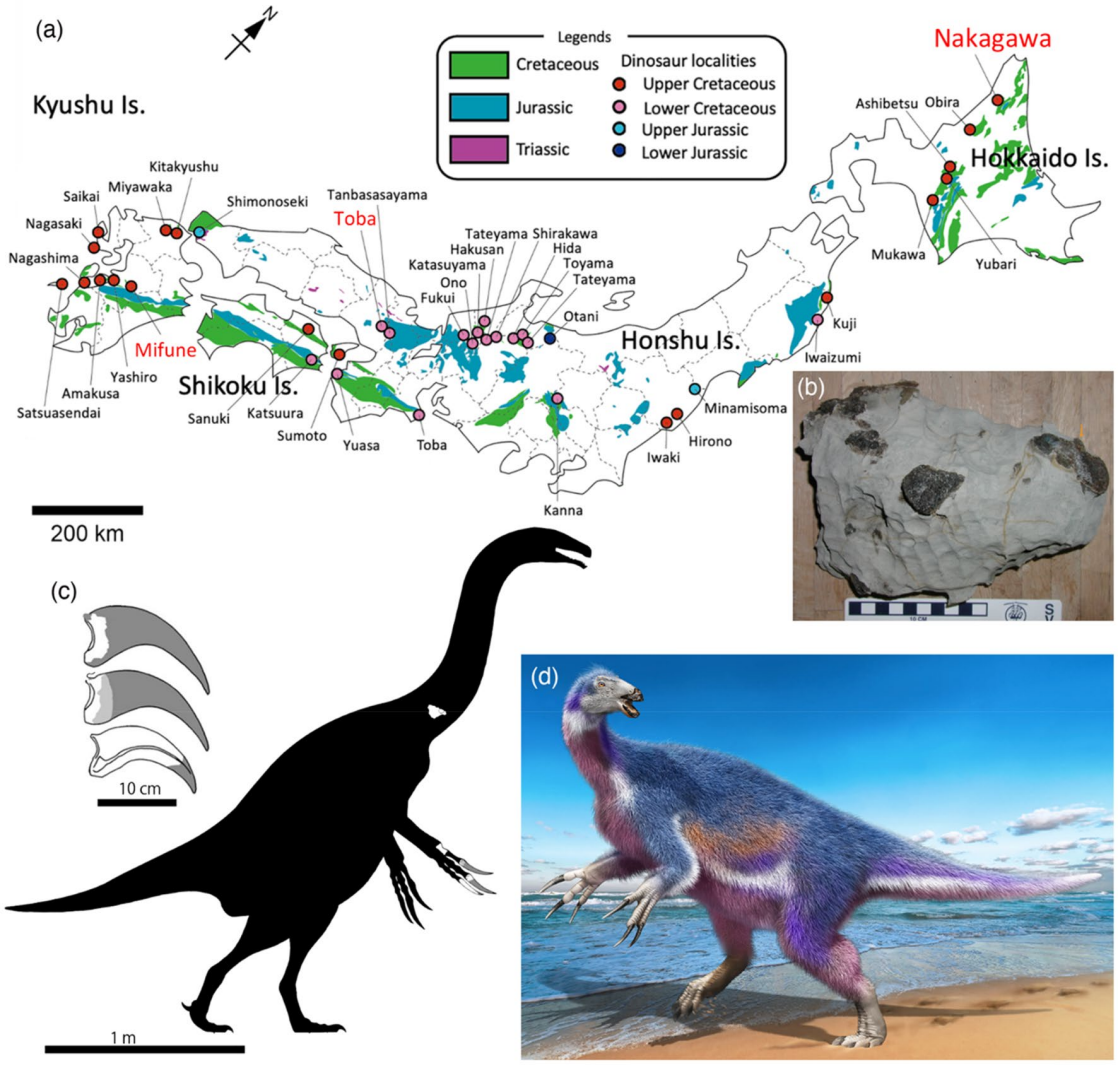
Map of Japan, showing the location of Nakagawa Town and other dinosaur localities (**a**). Two other names in red are locations of other therizinosaur materials. This figure was created by using Adobe Photoshop 21.2.0 and Adobe Illustrator 24.2.1 (https://www.adobe.com/). A photo of the concretion containing materials of *Paralitherizinosaurus* *japonicus* gen. et sp. nov. before preparation (**b**). Dark parts in the concretion are exposed bones of *Paralitherizinosaurus*, showing that all elements were preserved in this block. See Murakami et al.^1^ for stratigraphic column of the Oshoushinai Formation and the horizon of the specimen. Manual unguals and silhouette of *Paralitherizinosaurus japonicus*, showing recovered skeletal elements in white (**c**) (Courtesy of Genya Masukawa). Life reconstruction of *Paralitherizinosaurus japonicus* (**d**) (Courtesy of Masato Hattori).

In 2008, Murakami et al.^1^ described a partial manus of a theropod dinosaur, consisting of metacarpal I and manual unguals I-2, II-3, and III-4 from the right side in a concretion, from the upper Campanian Osoushinai Formation of the Yezo Group in Nakagawa Town of Hokkaido Island of Japan (Fig. 1a–c). The Yezo Group is composed of mainly Upper Cretaceous marine sediments and rich in invertebrate and vertebrate fossils, including sharks, plesiosaurs, mosasaurs, turtles, pterosaurs, non-avian dinosaurs, and birds. So far, dinosaur materials of hadrosaurids, tyrannosauroid, and nodosaurid, in addition to the Nakagawa theropod, have been reported from the group^2–4^. The Nakagawa specimen was identified as a maniraptoran dinosaur and Murakami et al.^1^ argued further that it may belong to a derived therizinosaur because of the lack of a groove between the flexor tubercle and the proximal articular surface of the unguals. It was extremely difficult to resolve the identification of Nakagawa specimen to the family level or lower at the time of publication because of the limited comparative information in the literature. However, more recent studies have provided a great deal of information on therizinosaur unguals, permitting comparisons of manual morphologies among therizinosaurs and for testing the phylogenetic status of the Nakagawa specimen^5–10^.

Asian therizinosaurs radiated in the Early Cretaceous, and their diversification continued into the Late Cretaceous. The Late Cretaceous taxa are larger than the Early Cretaceous forms in body size, exemplified by *Therizinosaurus cheloniformis* from Mongolia with elongate, large and nearly straight manual unguals. Manual unguals of therizinosaurs have a large diversity in shapes and functions. Based on the shape analysis with extinct and extant mammals by Lautenschlager^10^, basal therizinosaurs, such as *Alxasaurus* and *Erliansaurus*, have short and compact unguals for a proposed generalist functionality because these are placed near the boundaries of the morphospace of scansorial, fossorial, and terrestrial mammals, whereas other therizinosaurs with elongate unguals occupy outside of these mammals for potentially different functions. Only *Nothronychus* has unguals like those found in fossorial mammals. Because some therizinosaurs such as *Beipiaosaurus* and *Therizinosaurus*, have similar unguals to those of ornithomimosaurs, elongate unguals are presumed to have a similar function, possibly to pull the base of branches to bring leaves to their heads^10^.

Therizinosaurs have been found mainly from the Cretaceous deposits in Mongolia and China. All of the Early Cretaceous taxa, except *Falcarius*, have been named from China so far, and two taxa are recovered from northern (*Alxasaurus* from the Inner Mongolia)^11^ and northwestern (*Suzhousaurus* from Gansu Province) regions^12^. Other Chinese taxa (*Jianchangosaurus*, *Beipiaosaurus*, and *Lingyuanosaurus*)^6,13,14^ were recovered from the Jehol Group in the Liaoning Province of China, located in the eastern part of the country. The Late Cretaceous therizinosaurs have been discovered from the Gobi Desert (Inner Mongolia of China and southern Mongolia) except for *Nanshiungosaurus* (Guangdong Province in the southeastern China along the Pacific). Although it has not been named yet, therizinosaur materials have been recovered from the Bissekty Formation in Uzbekistan, which may represent more than two taxa^9^. However, we follow Sues and Averianov^9^ in their treatment of the Bissekty therizinosauroid material as a single taxon. Japan, which was located at the eastern edge of the Asian continent during the Cretaceous before the opening of the Japan Sea during the Miocene, has produced two therizinosaur specimens from the Lower and Upper Cretaceous deposits^15,16^ (Fig. 1a) but neither is named because of the fragmentary nature of the specimens. The Nakagawa specimen is the third therizinosaur from Japan. It is important because it is the youngest occurrence of therizinosaurs from Japan and preserves important elements that enlighten our understanding of its finer-scale taxonomic identification as well as providing insights into and morphological function.

This study will describe the Nakagawa material in detail, compare with other therizinosaurs, demonstrate its phylogenetic placement within Maniraptora/Therizinosauria, quantify the ungual shapes through geometric morphometric analysis to elucidate the evolution and function of therizinosaur claws, and discuss its paleogeographic and paleoecological implications.

### Geological setting

Detailed information is provided by Murakami et al.^1^ and summarized here. This specimen was contained in an isolated concretion as float, sitting near the confluence of the Rubeshibe River and 36 Ten-zawa Creek in Nakagawa Town in Hokkaido Prefecture, Japan (Fig. 1a). The concretion was discovered in an area where the Nishichirashinai Formation (Coniacian to lower Santonian) is exposed. Murakami et al.^1^ concluded that it was originally from an upstream area, where the Oshoushinai Formation (early Campanian) crops out because of the presence of the inoceramid bivalve *Sphenoceramus* in the concretion and the features of lithofaces (heavily bioturbated matrix and fine grain size), which matches the Osoushinai Formation. The depositional environment of this formation is considered as deeper than outer shelf because of the lack of storm deposits. The concretion is roughly 25 cm × 15 cm × 15 cm (Fig. 1b). It is plausible to consider that all materials belong to a single individual based on its depositional environment and close proximity of all preserved elements as mentioned by Murakami et al.^1^.

### Systematic paleontology

Theropoda Marsh^17^.

Coelurosauria von Huene^18^.

Therizinosauria Russell^19^.

Therizinosauridae Maleev^20^.

*Paralitherizinosaurus japonicus* gen. et sp. nov.

Zoobank ID: urn:lsid:zoobank.org:pub:8397F6AE-4791-4EE6-B6CF-B25950FB617C (for this publication), urn:lsid:zoobank.org:act:7934F3DA-B1CA-4CAA-B5E4-86D41B93E7CE (for the new genus) and urn:lsid:zoobank.org:act:BC19DCA2-8F4A-4FDC-83C4-B9EB8D3C30EF (for the new species).

*Etymology* *“Paralos”* means by or near the sea in Greek, “*therizo”* means reap in Greek, and “*sauros*” means reptile in Latin. Specific name, “*japon*” refers to Japan.

*Holotype* NMV-52 (NMV, Nakagawa Museum of Natural History, Japan), a partial vertebra and a partial right hand, including metacarpal I, proximal ends of unguals I and II, and nearly complete ungual III (Fig. 1c).

*Horizon and locality* The Osoushinai Formation (early Campanian) of the Yezo Group in Nakagawa Town in Hokkaido Prefecture, Japan.

*Diagnosis* A therizinosaurid with the following unique characters: dorsoventrally flattened metacarpal I (dorsoventral height less than half of transverse width) with no rotation of the axis of the distal end; presence of proximodorsally projecting dorsal lip in digits I and III; a shallow depression at the proximal portion of ungual III-4, connecting to the collateral groove; the presence of proximally extending ventral process; a weak flexor tubercle expressed as a small ridge.

**Figure 2:**
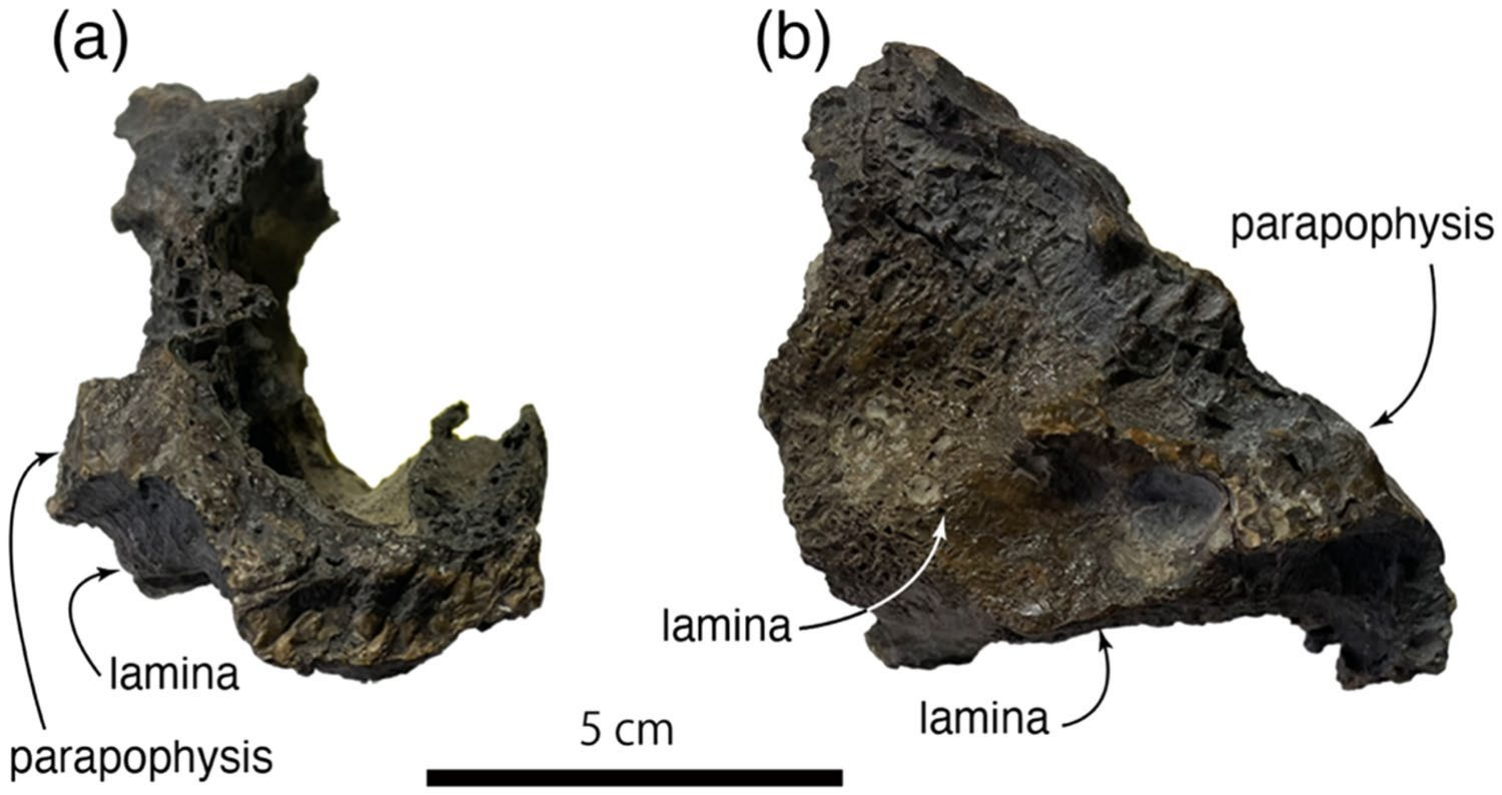
A partial vertebra of *Paralitherizinosauruss japonicus* gen. et sp. nov. in anterior (**a**) and right lateral (**b**) views. This figure was created by using Adobe Photoshop 21.2.0 and Adobe Illustrator 24.2.1 (https://www.adobe.com/).

*Description and comparisons* The original study described four manual elements from the right side (metacarpal I and manual unguals I-2, II-3, and III-4), but this study identified another bone as a partial cervical centrum (Fig. 2) although the fragmentary nature of this bone limits the certainty of its identification. We interpret the specimen as the anteroventral portion of a cervical centrum. Therizinosaur presacral vertebrae are highly pneumatic^8,14,21^, a condition that is present in this specimen. The parapophysis is ventrally placed on the lateral surface. Two laminae, posterodorsal and ventral laminae, extend posterior to the parapophysis. The arrangement of the posterodorsal lamina is similar to the centrodiapophyseal lamina in *Northonychus*^8^. Ventral surface is flat and featureless.

**Figure 3:**
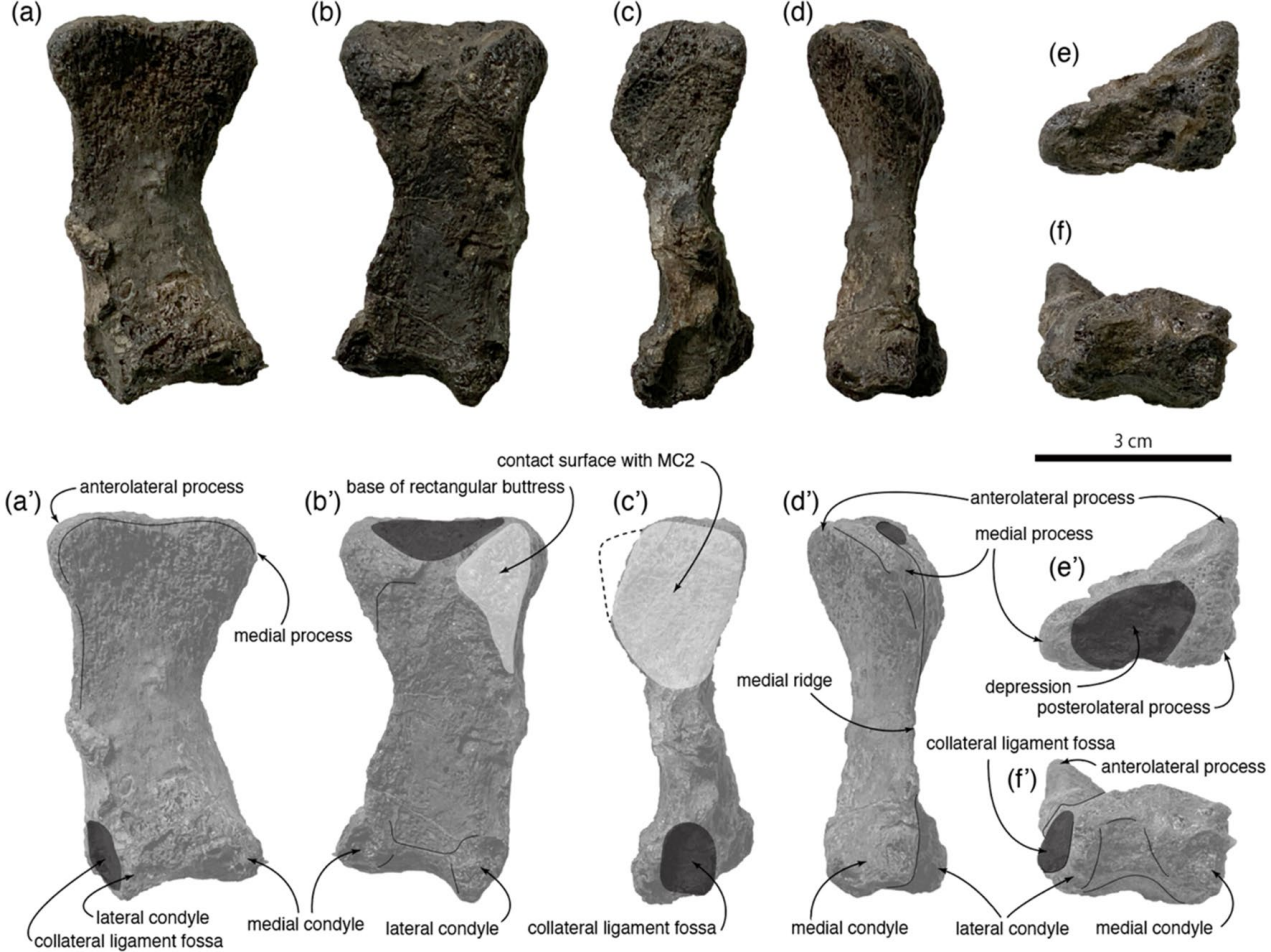
Right metacarpal I of *Paralitherizinosauruss japonicus* gen. et sp. nov. in anterior (**a**), posterior (**b**), lateral (**c**), medial (**d**), proximal (**e**), and distal (**f**) views. (**a′**) to (**f′**) are corresponding images in black and white with labels. This figure was created by using Adobe Photoshop 21.2.0 and Adobe Illustrator 24.2.1 (https://www.adobe.com/).

Metacarpal I is nearly complete. It is 57.87 mm long, which is approximately twice the length of the transverse width (29.41 mm) at the proximal end. In anterior view, the distal half of metacarpal I diverges medially by 17 degrees from the contact surface with metacarpal II (Fig. 3a,a′,c,c′). A similar condition is present in two Chinese therizinosaurids (*Alxasaurus* and *Erliansaurus*), while the shaft of metacarpal I is nearly straight in basal therizinosauroids (*Falcarius* and *Jianchangosaurus*) and the therizinosaurid *Therizinosaurus*^5,14,22^. The shaft width (21.72 mm) is roughly two-thirds of the proximal width. The medial border of this element is strongly concave in anterior view (Fig. 3a,a′), whereas it is nearly straight in *Nothronychus* and *Therizinosaurus* and weakly concave in *Falcarius*, *Alxasaurus*, and *Erliansaurus*^5,8,11,22,23^. The width of the proximal end is greater than the dorsoventral height (24.47 mm). In proximal view, the proximal end is triangular with three processes: medial, anterolateral, and posterolateral processes. In their preliminary description of this specimen, Murakami et al.^1^ noted that the presence of a rectangular buttress was not clear because of its preservation. Our examination shows that the base of the ventrolateral process is preserved, and a ridge extends ventrally from the process, indicating the presence of a rectangular buttress (Fig. 3b,b′,c,c′). The buttress would underlie the ventral surface of the metacarpal II if entirely preserved, and this is a synapomorphic feature for the clade of Therizinosauroidea^11^. In anterior view, the proximal edge of the proximal end between the dorsal and mediolateral processes is straight as in therizinosaurids (e.g., *Alxasaurus* and *Therizinosaurus*). *Falcarius* has a concave proximal edge of the proximal end for a dorsomedial notch^22^. The proximal surface has a depression with a size of 21 mm × 14 mm (Fig. 3e,e′). The medial edge of the element bears a medial ridge (Fig. 3d,d′), connecting the medial process of the proximal end and medial condyle of the distal end. This feature is present only in *Therizinosaurus* and diagnostic for this taxon^5^. The cross-section of the shaft is triangular as in *Therizinosaurus*, whereas it is oval in *Falcarius*^22^. It is anteroposteriorly compressed, and its dorsoventral height is 10.12 mm, less than half of lateromedial width of the shaft. A rotation of the axis of distal end from the long axis of the element is noted in *Falcarius*^22^ and *Erliansaurus*, but this rotation is absent in *Paralitherizinosaurus*. The distal end has lateral and medial condyles, separated by a shallow sulcus (Fig. 3f,f′). The lateral condyle has a circular collateral ligament fossa, whereas the medial condyle lacks a fossa and has a flat medial surface. A collateral ligament fossa is absent in *Falcarius*^22^ and *Therizinosaurus*. The medial condyle is proximally positioned with respect to the lateral condyle in therizinosaurs, but this condition is more subtle in *Paralitherizinosaurus*.

**Figure 4:**
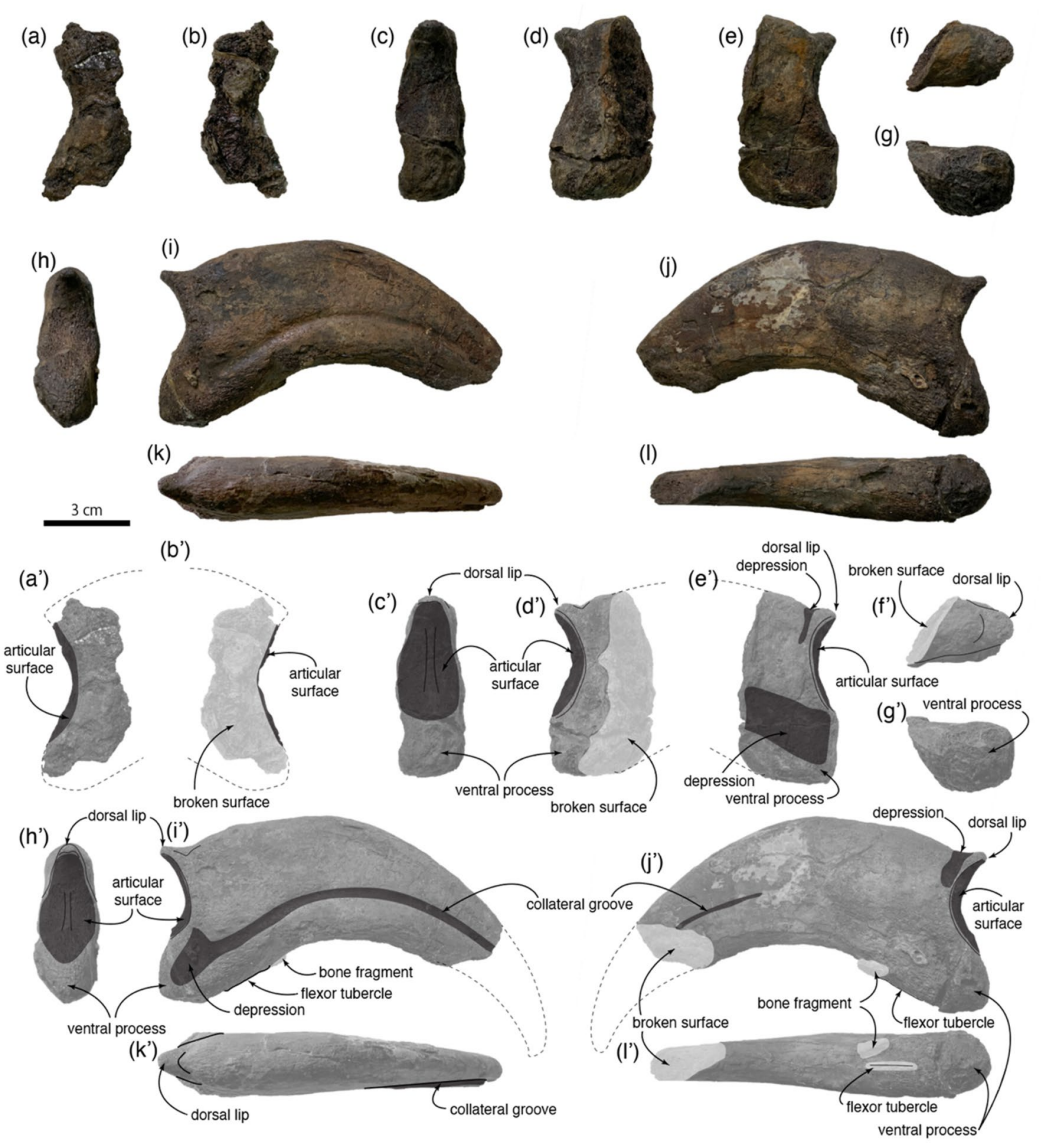
Right manual unguals of *Paralitherizinosauruss japonicus* gen. et sp. nov. Ungual of digit I in lateral (**a**) and medial (**b**) view. Ungual of digit II in proximal (**c**), lateral (**d**), medial (**e**), dorsal (**f**), and ventral (**g**) views. Ungual of digit III in proximal (**h**), lateral (**i**), medial (**j**), dorsal (**k**), and ventral (**l**) views. (**a′**) to (**l′**) are corresponding images in black and white with labels. This figure was created by using Adobe Photoshop 21.2.0 and Adobe Illustrator 24.2.1 (https://www.adobe.com/).

Manual ungual I-2 preserves only lateral side of the proximal end (Fig. 4a,b,a′,b′). It has a dorsal lip, which is a proximodorsally projecting process above the phalangeal articular surface of manual unguals. This process is commonly seen in manual unguals of therizinosaurs, oviraptorosaurs, and dromaeosaurids^24^. In derived therizinosaurs, Therizinosauridae, no taxa have a dorsal lip in manual ungual I-2. The only therizinosaur with a dorsal lip is the basal therizinosaur *Beipiaosaurus*^13^. In lateral view, the outline of the surface is like the other manual unguals of *Paralitherizinosaurus* (Fig. 4a,a′).

**Figure 5:**
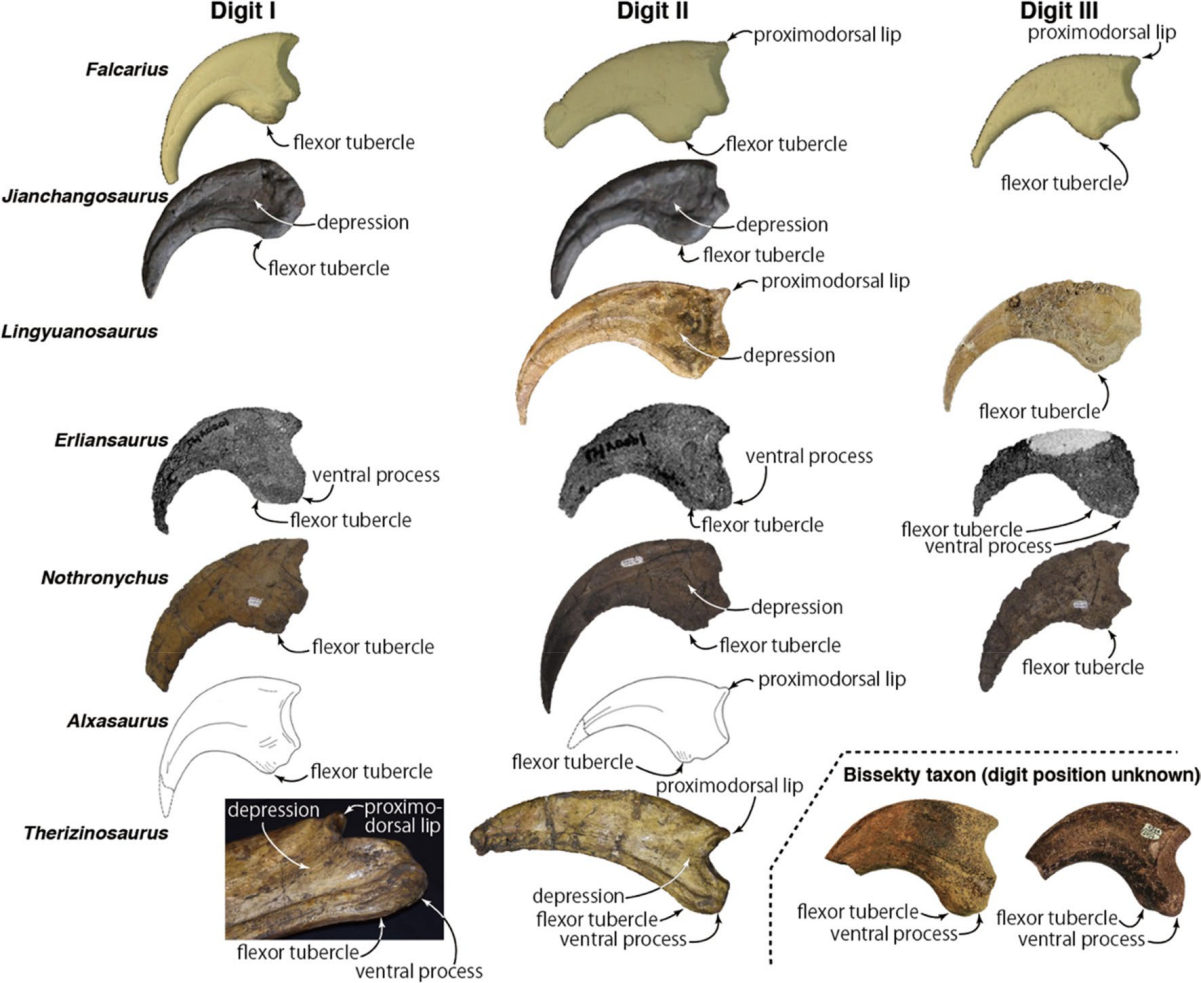
Comparisons of manual unguals in digits I-III of therizinosaurs. An image at the lower left corner is a photo of the proximoventral end of *Therizinosaurus* ungual in oblique view. Not to scale. This figure was created by using Adobe Photoshop 21.2.0 and Adobe Illustrator 24.2.1 (https://www.adobe.com/).

Manual ungual II-3 preserves the proximal portion of the element and exhibits a pronounced dorsal lip as seen in *Falcarius*, *Lingyuanosaurus*, *Alxasaurus*, and *Therizinosaurus* (Figs. 4d–f,d′–f′ and Fig. 5)^6,11,22,25^. The ventral surface of this lip forms a dorsal portion of the phalangeal articular surface. The medial side of the base of the lip has a shallow depression, extending ventrally (Fig. 4e,e′). The ventral half of the medial surface has a wide depression. A depression on lateral and medial surfaces of proximal portions in *Jianchangosaurus, Lingyuanosaurus,* and *Therizinosaurus* is continuous from the collateral groove^6,14,25^. Ventral to the phalangeal articular surface bears a ventral process, which extends slightly more proximally than the articular surface, similar to the condition observed in *Erliansaurus* (Fig. 4e,e′,g,g′). It is square in lateral view as in *Therizinosaurus*. The phalangeal articular surface is divided asymmetrically by a vertical ridge, and the medial side is larger than the lateral side (Fig. 4c,c′).

Manual ungual III-4 is transversely narrow as in other unguals (Fig. 4k,k′,l,l′) and strongly curved as in other therizinosaurs, other than *Therizinosaurus*^5,25^ (Fig. 4i,i′,j,j′). The collateral groove on the lateral surface of this element approaches the dorsal margin of the ungual distally. Although the distal end is missing, the collateral groove may be extended to the dorsal edge of the distalmost part of the ungual in *Paralitherizinosaurus* (Fig. 4i,i′), which is a potential diagnostic feature for Therizinosauroidea^6^. The groove at the proximal end is close to the ventral edge of the ungual, like the ungual of digit II of *Therizinosaurus* and isolated unguals from the Bissekty taxon (Fig. 5), and is continuous with a shallow depression. This depression is much smaller than the ones observed in *Jianchangosaurus*, *Lingyuanosaurus*, *Nothronychus*, and *Therizinosaurus*^6,8,14^, which have a large triangular depression. Extension of the collateral groove to the proximal end is only seen in isolated unguals of the Bissetky taxon (Fig. 5). The medial surface of the ungual is flat and featureless (Fig. 4j,j′). Distally, the collateral groove is faintly present and migrates dorsally towards its tip. In most therizinosaurs, both the lateral and the medial surfaces have distinct collateral grooves. The asymmetry of features on the lateral and the medial surfaces is present to some extent, but this strong asymmetry may be a unique feature for *Paralitherizinosaurus.* The ungual has a dorsal lip at the proximal end (Fig. 4i,i′,j,j′). In Therizinosauria, a complete set of manual unguals is rarely preserved but has been reported in five taxa (*Falcarius*, *Beipiaosaurus*, *Martharaptor*, *Erliansaurus,* and *Nothronychus*). Among these taxa, only two taxa have a dorsal lip, which is present in digits II and III in *Falcarius* and in digits I in *Beipiaosaurus*. Three therizinosaurids (*Lingyuanosaurus*, *Alxasaurus,* and *Nothronychus*) preserve the manual ungual of digit III, but none of these taxa preserves a dorsal lip because of damage, suggesting the presence of a dorsal lip in manual ungual III-4 may be unique to *Paralitherizinosaurus*. At the base of the dorsal lip is a shallow depression as in the ungual of digit II. The phalangeal articular surface is divided by a vertical ridge, but it is nearly symmetrical (Fig. 4h,h′). Ventral to the articular surface has a ventral process, which extends more proximally than the articular surface. This large ventral process is present in the ungual of the digit III of *Erliansaurus*. The ventral surface of the ventral process bears a weak ridge, which is a flexor tubercle. The original description of this ungual interpreted that the flexor tubercle was missing because of bioerosion^1^. Subsequent preparation of this ungual shows that this portion was not damaged and there is an extremely weak flexor tubercle on the ventral surface (Fig. 4l,l′). A similar condition is present in *Therizinosaurus* (Fig. 5), where the other therizinosaurs have a strong flexor tubercle.

## Discussion

**Figure 6:**
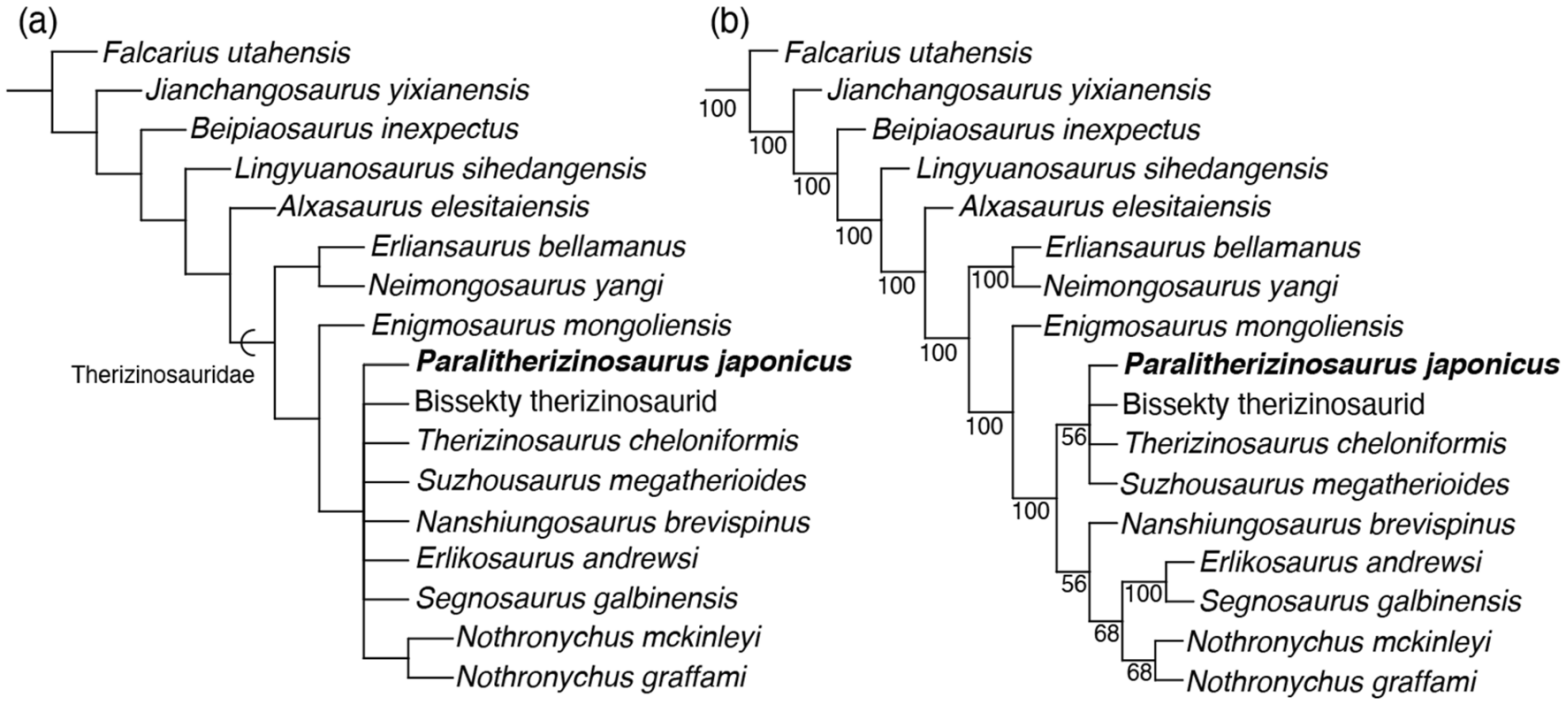
Strict consensus tree (**a**) and 50% majority-rule consensus tree (**b**) of Therizinosauria in this study. This figure was created by using Adobe Illustrator 24.2.1 (https://www.adobe.com/).

A phylogenetic analysis in this study recovered 970 most parsimonious trees with 1294 steps. The consistency index is 0.349, and the retention index is 0.702 for these trees. The strict consensus tree places *Paralitherizinosaurus* within the unresolved clade, consisting of derived therizinosaurs such as *Therizinosaurus*, *Nothronychus*, *Nanshiungosaurus*, *Suzhousaurus*, *Erlikosaurus*, and *Segnosaurus* (Fig. 6a). This clade is supported by four synapomorphies (cranially focused pubic boot with little to no caudal process [character 178], ischiadic peduncle of ilium and antitrochanter forming hypertrophied and spherical boss [character 312], mediolaterally flattened pubic shaft [character 317], greatly enlarged distal pubic shaft [character 318]). According to taxonomic definitions by Zanno^5^, this clade is named as Therizinosauridae; however, members of this clade are not stable because of poor resolution for derived therizinosaurs. For instance, Bissekty taxon^6,9^, *Erliansaurus*^14^, *Nanshiungosaurus*^7,14^, *Neimongosaurus*^14^, and *Suzhousaurus*^7^ are excluded from the clade of Therizinosauridae based on the definition by Zanno^5^. A new definition of Therizinosauridae is proposed here for the stability of this clade: the most inclusive clade containing *Therizinosaurus cheloniformis* but not *Alxasaurus elesitaiensis*. This clade is well supported by nine synapomorphies (extensive axial pneumaticity extending through more than half of dorsal vertebrae [character 106], six sacral vertebrae [character 110], rostrally edentulous dentary [character 220], significantly expanded distal humerus [character 293], ginglymoid distal end of metacarpal III in dorsal view [character 296], weakly developed or absent ligament pits on manual phalanges [character 302], strongly curved pedal unguals III and IV [character 337], expanded medial aspect of distal humerus and subtriangular in cranial view with entepicondyle located well medial to ulnar condyle [character 343], ventral process ventral to the articular surface of manual unguals [character 355]). Based on this definition, all members of non-therizinosaurid therizinosaurs are recovered from the Early Cretaceous whereas all therizinosaurids except for *Suzhousaurus* are found from the Late Cretaceous.

The monophyly of *Erliansaurus* and *Neimongosaurus* is posited as the most basal clade of Therizinosauridae, and *Enigmosaurus* is a sister taxon to the unresolved clade of derived therizinosaurids. The 50% majority-rule consensus tree shows better resolution within derived therizinosaurids (Fig. 6b). *Therizinosaurus*, *Suzhousaurus*, Bissekty taxon, and *Paralitherizinosaurus* form a monophyletic clade, supported by three synapomorphies (dorsal flange at dorsal margin of scapular blade [character 284], weak flexor tubercles of manual unguals [character 353], and lateral and medial grooves of manual unguals extending to proximal end [character 356]), but their relationships are unresolved. The clade of *Nanshiungosaurus* and higher taxa is supported by a single character (ischiadic peduncle of ilium and antitrochanter forming hypertrophied and spherical boss [character 312]), and that of *Nothronychus*, *Erlikosaurus*, and *Segnosaurus* share a single character (fused sacral neural spines into continuous spinal ridge [character 278]). It is noteworthy that two therizinosaurids (*Erlikosaurus* and *Segnosaurus*) from the same formation form a clade by sharing another character (trochanter or crest on caudomedial surface of humeral shaft [character 287]). *Paralitherizinosaurus* shows an affinity with *Falcarius* in having a dorsal lip in unguals of digits II and III; with *Lingyuanosaurus* in having a dorsal lip in ungual of digit II and a shallow depression on the lateral surface; with *Erliansaurus* in having a ventral process at the proximal end; with *Nothronychus* in having a shallow depression on the lateral surface; with *Alxasaurus* in having a dorsal lip in ungual of digit II and ventrally positioned collateral groove; with *Therizinosaurus* in having a dorsal lip in ungual of digit II, a shallow depression on the lateral surface, a ventral process at the proximal end, extremely weak flexor tubercle, and ventrally positioned collateral groove.

**Figure 7:**
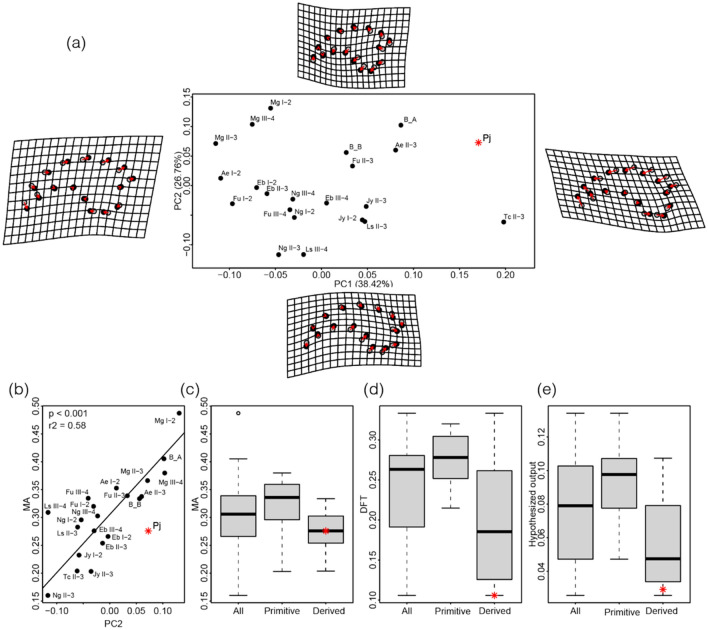
(**a**) Scatter plot showing the PC1 and PC2 values obtained from the geometric morphometric analysis. The grey dots on the thin plate spline^26^ represent the “average” positions of the landmarks and the black dots represent the positions after deformation. (**b**) Regression plot of PC2 values obtained from the geometric morphometric analysis and the mechanical advantage of the therizinosauroid unguals. Boxplots showing the (**c**) mechanical advantages, (**d**) development of flexor tubercle, and (**e**) hypothesized output force among therizinosauroids. The red star represents *Paralitherizinosaurus japonicus* in all plots. Abbreviations: Ae: *Alxasaurus elesitaiensis*, B_A: Bissekty taxon A, B_B: Bissekty taxon B, Eb: *Erliansaurus bellamanus*, Pj: *Paralitherizinosaurus japonicus*, Fu: *Falcarius utahensis*, Jy: *Jianchangosaurus yixianensis*, Ls: *Lingyuanosaurus sihedangensis*, Mg: *Martharaptor greenriverensis*, Ng: *Nothronychus graffami*, Tc: *Therizinosaurus cheloniformis*. This figure was created by using Adobe Photoshop 21.2.0 and Adobe Illustrator 24.2.1 (https://www.adobe.com/).

In this study, geometric morphometrics analysis of therizinosaur manual unguals demonstrates that the first two principal components (PC1 and PC2) explain > 65% of the total shape variation (Supplementary Table S1). In PC1, high values indicate a ventrodistal shift of the proximodorsal border, a dorsodistal shift of ventrodistal border, and a proximal shift of proximoventral border from the mean shape, resulting in the ungual elongation. *Therizinosaurus* has the highest PC1 value (0.20), followed by *Paralitherizinosaurus* (0.17) (Fig. 7a). Most of the therizinosaurs fall within the range of -0.10 and 0.10, whereas *Paralitherizinosaurus* and *Therizinosaurus* are far outside of the range. In PC2, high values suggest a dorsodistal shift of proximodorsal border, a ventrodistal shift of proximoventral border, and a distal shift of the phalangeal articular surface, resulting in a robust ungual with a larger articular surface and a pronounced flexor tubercle. *Paralitherizinosaurus* has a higher PC2 value (0.07) than *Therizinosaurus* (− 0.06). The mechanical advantage (MA) of unguals shows little correlation with PC1 (p > 0.05) but a statistically significant correlation with PC2 (Fig. 7b). A residual of *Paralitherizinosaurus* from the regression line is negatively the largest among therizinosaurs. *Paralitherizinosaurus* has a similar PC2 value to the unguals of digit II of *Martharaptor* (0.07) and *Alxasaurus* (0.06), but MA of *Paralitherizinosaurus* (0.28) is as low as *Erliansaurus* (0.25–0.28). Boxplots of MAs of therizinosaurs show that derived therizinosaurs (therizinosaurids) tend to have smaller values of MA than primitive therizinosaurs (non-therizinosaurid therizinosaurs) although the difference is statistically insignificant (Welch’s test, p = 0.17). *Paralitherizinosaurus* is plotted near the median value of MA for derived therizinosaurs (Fig. 7c). Derived therizinosaurs tend to have smaller flexor tubercles than primitive therizinosaurs (Fig. 7d). *Paralitherizinosaurus* has the least development of flexor tubercle (DFT) (0.11), followed by *Erliansaurus* (0.12) and *Therizinosaurus* (0.13), among therizinosaurs. If DFT is a proxy of the degree of force input, hypothesized outputs (product of MA and DFT) of derived therizinosaurs are less than those of primitive therizinosaurs (Fig. 7e). *Paralitherizinosaurus* has the second smallest value of hypothesized output (0.029), whereas *Therizinosaurus* has the smallest (0.026). In addition, the posteroventrally expanded ventral process of *Paralitherizinosaurus* and *Therizinosaurus* contacts the preceding phalanx in flexion by a lower degree of the range of motion, thus expected to limit the ungual mobility. This small hypothesized output value and the limited range of motion may together indicate that the unguals in these taxa were less functional compared to the basal taxa. These results also show a possible evolutionary trend in decreases of MA, DFT, and hypothesized outputs among therizinosauroids. The combination of this study and the previous study by Lautenschlager^10^ suggest that primitive therizinosaurs have unguals with generalist functionalities and that the unguals of derived therizinosaurs did not function with a strong force such as scansorial, fossorial, or terrestrial behaviors but was stiffer as a rake for hook-and-pull function to bring vegetation to the head^24^.

*Paralitherizinosaurus* is the third therizinosaur specimen from Japan^4^, following a single tooth from the Lower Formation of the Sasayama Group (early Albian?)^15^ and a partial braincase, teeth, and humerus from the Upper Formation of the Mifune Group (Cenomanian-Coniacian?) in Kyushu Island^16^. Although neither specimen has been described yet, it has been suggested that the Sasayama therizinosaur was similar to the Early Cretaceous *Falcarius* (Barremian) from Utah based on the size of denticles^15^ and the Mifune specimen showed affinities with *Erlikosaurus* from the Bayanshiree Formation (Cenomanian–Turonian) of Mongolia in tooth and humerus morphologies^16^. *Paralitherizinosaurus* is from the early Campanian Osoushinai Formation^1^, showing that it is the youngest occurrence of therizinosaur remains in Japan and a wide temporal distribution of therizinosaurs from the Early to Late Cretaceous at the eastern edge of the Asian continent^5^. The latest Cretaceous (Campanian to Maastrichtian) therizinosaurs in Asia are represented by *Erliansaurus*, *Neimongosaurus*, and *Nanshiungosaurus* from China and *Therizinosaurus* from Mongolia as well as *Paralitherizinosaurus*, indicating a wide longitudinal and latitudinal geographic distribution (more than 1000 km in both directions) of therizinosaurs in Asia^5^. It is noteworthy that *Paralitherizinosaurus* is the first therizinosaur record from marine sediments in Asia and the second taxon in the world. The first record from marine sediments was *Nothronychus graffami* from the Tropic Shale^27^. These occurrences from marine sediments suggest that some therizinosaurids, hadrosaurids, and nodosaurids were adapted to coastal environment (Fig. 1d) in both Asian and North American continents^2,28,29^.

## Materials and methods

### Phylogeny

A phylogenetic analysis was performed using TNT (Tree Analysis Using New Technology) v. 1.5^30^ and the data matrix of Yao et al.^6^ with the addition of the new therizinosaur described here. In addition, character 151 in Yao et al.^6^ is modified into three characters (151, 353, and 354) (Supplementary Text S2). The resultant matrix comprises 79 OTUs of 356 characters (Supplementary Data S3). Most parsimonious trees were obtained by heuristic search methods on 1000 replicates of Wagner trees with random addition sequences and subject to tree bisection-reconnection swapping methods holding 10 trees per replicate. As noted by Yao et al.^6^, twenty-one characters (characters 27, 37, 40, 68, 76, 78, 97, 106, 113, 157, 163, 168, 253, 303, 304, 308, 309, 310, 334, 342, and 345) were designated additive and two characters (characters 165 and 215) are excluded.

### Definition of taxonomic names

This study follows Zanno^5^ for the definition of Therizinosauria, which is the most inclusive clade containing *Therizinosaurus cheloniformis* but not *Tyrannosaurus rex*, *Ornithomimus edmontonicus*, *Mononykus olecranus*, *Oviraptor philoceratops* or *Troodon formosus*. Zanno et al.^27^ defined Therizinosauridae as the least inclusive clade containing *Nothronychus*, *Segnosaurus galbinensis*, *Erlikosaurus andrewsi*, and *Therizinosaurus cheloniformis*, but this study redefined this group as the most inclusive clade containing *Therizinosaurus cheloniformis* but not *Alxasaurus elesitaiensis* as discussed in the main text.

### Geometric morphometric analysis

To quantify two-dimensional ungual shape variations, geometric morphometric analysis was performed using R package geomorph version 3.3.1.^31^. Ungual images other than of *Paralitherizinosaurus* and *Therizinosaurus* were obtained from the literature^6–9,11,14,22,23^. Outlines of the unguals in the lateral view were digitalized into four fixed landmarks and 12 sliding semi-landmarks (Supplementary Datas S4–6), following Chinzorig et al.^32^. The landmarks were subjected to the generalized Procrustes analysis^33,34^ to superimpose the specimens. This analysis first scales all shapes (ungual landmarks in this case) into the same size, then rotates the shape coordinates around the origin until the differences among the shapes are minimized. Using the covariance matrix of the Procrustes coordinates, the principal component analysis (PCA) was performed. PCA finds the maximum variance in multidimentional (multi-variable) dataset that summarizes the original data as PC1, the following variance as PC2, and so on. As the result, PCA allows graphical illustration of multivariate data into two-dimensional graph as shown in Fig. 7^35,36^. To infer the functionality of therizinosaur unguals, the mechanical advantage (MA) was calculated. Since MA is equivalent to a class 3 lever^37^, resultant MA values represent the proportion of the output force applied on the tip of the ungual relative to the input force at the flexor tubercle. The mechanical advantage of the ungual can be calculated as:

1 $${\text{MA }} = \, \sin \left( {\theta \, + \, \delta } \right){\text{ d}}/{\text{a}}$$

In the Eq. (1), a is the output lever length from the point of the fulcrum to the resistance, d is the length from the point of the fulcrum to the flexor tubercle, θ is the angle of the input force vector to the line of output lever, and δ is the angle between the line from the point of the fulcrum to the flexor tubercle and the line of output lever^32^ The flexor tubercle size is considered to be closely related to the cross-sectional area of the attached muscle, which should correspond to the maximum input force. Therefore, flexor tubercle size was quantified as a ratio of the perpendicular length from the apex of the flexor tubercle to the segment between the base of the flexor tubercle as a proxy of the input force. The flexor tubercle size was then multiplied by the mechanical advantage to infer the output force at the tip. The inferred output forces were compared among digits (I, II, and III) and taxonomically (non-therizinosaurid therizinosaurs and therizinosaurids). Standardized major axis (SMA) regression analyses were conducted using R package smatr version 3.4.8 to test the relationship between the obtained PC scores and the inferred output and the inferred output force to test the shape-function relationships of the therizinosaur unguals. All of the statistical analyses were conducted on software R version 4.0.2^38^. The analyses are conducted using the R script provided as Supplementary Data S6.

## Supplementary Information

Supplementary Table S1.

Supplementary Information 2.

Supplementary Information 3.

Supplementary Information 4.

Supplementary Information 5.

Supplementary Information 6.

Supplementary Information 7.

## Data Availability

All data generated or analysed during this study are available as supplementary information files at figshare (https://doi.org/10.1038/s41598-022-11063-5).
