# Supplementary material for: New therizinosaurid dinosaur from the marine Osoushinai Formation (Upper Cretaceous, Japan) provides insight for function and evolution of therizinosaur claws
Source: Sci Rep. 2022 May 3;12:7207. doi: 10.1038/s41598-022-11063-5 (PMC9065154; doi:10.1038/s41598-022-11063-5)
Supplement: Supplementary file 2 — Supplementary Information 2. [file 41598_2022_11063_MOESM2_ESM.docx]

Supplementary Data S2. List of characters used in our phylogenetic analysis.

1. Vaned feathers on forelimb symmetric (0) or asymmetric (1).

## **Skull**

2. Orbit round in lateral or dorsolateral view (0) or dorsoventrally elongate (1).

3. Rostral process of postorbital projects into orbit (0) or does not project into orbit (1).

4. Postorbital in lateral view with straight rostral (frontal) process (0) or frontal process curves rostrodorsally and dorsal border of temporal bar is dorsally concave (1).

5. Postorbital bar parallels quadrate, lower temporal fenestra rectangular in shape (0) or jugal and postorbital approach or contact quadratojugal to constrict lower temporal fenestra (1).

347. Craniocaudal width of postorbital bar subequal to preorbital bar (0) or expanded,

greater than twice the width of preorbital bar (1).

6. Enlarged pneumatic recess on lateral wall of braincase absent (0) or a well developed crescent shaped crest forms rostral edge of large rostral (anterior) tympanic recess excluding subotic recess if present (1) or crest delineates rostral and dorsal margins of a depression housing both the rostral tympanic and subotic recesses (lateral depression sensu lato) (2).

344. Cranial (anterior) tympanic recess confluent with subotic recess (lateral depression sensu lato) (0) lateral depression encompasses otic recess (lateral depression sensu stricto) (1).

7. Crista interfenestralis confluent with lateral surface of prootic and opisthotic (0) or distinctly depressed within middle ear opening (1).

224. Depression (possibly pneumatic) on ventral surface of postorbital process of laterosphenoid absent (0) or present (1). (Makovicky *et al.* 2003)

8. Subotic recess (pneumatic fossa ventral to fenestra ovalis) absent (0) or present (1).

253. Basisphenoid without (0) or with (1) inflated basisphenoidal bulla.

254. Basitubera well developed (0) or reduced (1) or absent (2).

9. Basisphenoid recess present between basisphenoid and basioccipital (0) or entirely within basisphenoid (1) or absent (2).

10. Caudal opening of basisphenoid recess single (0) or divided into two foramina or fossae by a thin bar of bone (1).

11. Base of cultriform process not highly pneumatized (0) or base of cultriform process (parasphenoid rostrum) expanded and pneumatic (parasphenoid bulla present) (1).

225. Basal tubera set far apart, level with or beyond lateral edge of occipital condyle and/or foramen magnum (may be connected by a web of bone or separated by a large notch) (0) or tubera small, directly below condyle and foramen magnum, and separated by a narrow notch (1). (Makovicky *et al.* 2003)

12. Basipterygoid processes ventral or rostroventrally projecting (0) or lateroventrally projecting (1).

13. Basipterygoid processes well developed, extending as a distinct process from the base of the basisphenoid (0) or processes abbreviated or absent (1).

14. Basipterygoid processes solid (0) or processes hollow (1).

15. Basipterygoid recesses on dorsolateral surfaces of basipterygoid processes absent (0) or present (1).

16. Depression for pneumatic recess on prootic absent (0) or present as dorsally open fossa on prootic/opisthotic (1) or present as deep, posterolaterally directed concavity (2).

17. Accessory tympanic recess dorsal to crista interfenestralis absent (0) small pocket present (1) or extensive with indirect pneumatization (2).

18. Caudal (posterior) tympanic recess absent (0) present as opening on rostral surface of paroccipital process (1) or extends into opisthotic posterodorsal to fenestra ovalis, confluent with this fenestra (2).

19. Exits of C. N. X-XII flush with surface of exoccipital (0) or cranial nerve exits located together in a bowl-like depression (1).

252. Subcondylar recess absent (0) or subcondylar recess present and isolated from exits of CN X-XII (1) or subcondylar recess and cranial nerves exit together in a deep depression encompassing multiple pneumatic fossae and enclosed by a well developed rim (2).

256. Supraoccipital with pronounced sagital crest (0) or crest reduced or absent (1).

20. Maxillary process of premaxilla contacts nasal to form caudal border of nares (0) or maxillary process reduced so that maxilla participates broadly in external naris (1) or maxillary process of premaxilla extends caudaly to separate maxilla from nasal posterior to nares (2).

21. Internarial bar rounded (0) or flat (1).

22. Crenulate margin on buccal edge of premaxilla absent (0) or present (1).

23. Caudal margin of naris farther rostral than (0), or nearly reaching or overlapping (1), the rostral border of the antorbital fossa. (Chiappe *et al.* 1998)

24. Premaxillary symphysis acute, V-shaped (0) or rounded, U-shaped (1).

25. Secondary palate short (0) or long, with extensive palatal shelves on maxilla (1).

26. Palatal shelf of maxilla flat (0) or with midline ventral ‘tooth-like’ projection (1).

238: Jugal process of maxilla, ventral to the external antorbital fenestra dorsoventrally narrow (0) or dorsoventrally wide (1). (modified from Senter *et al.* 2004:char. 14)

240: Nasal process of maxilla, dorsal ramus (ascending ramus of maxilla): prominent, exposed medially and laterally (0) or absent or reduced to slight medial, and no lateral exposure (1). (modified from Gauthier, 1986 and Cracraft, 1986 by Chiappe, 1996:char. 6)

244: In lateral view, lateral lamina of the ventral ramus of nasal process of maxilla: present, large broad exposure (0) or present, reduced to small triangular exposure (1).

241: In lateral view, participation of the ventral ramus of the nasal process of the maxilla in the rostral margin of the internal antorbital fenestra: present extensively (0) or small dorsal projection of the maxilla participates in the rostral margin (1) or no dorsal projection of maxilla participates in the rostral margin (2). (modified from Clarke & Norell 2002: char. 11)

242: In lateral view, dorsal border of the internal antorbital fenestra formed by lacrimal and maxilla (0) or by lacrimal and nasal (1).

243: In lateral view, dorsal border of the antorbital fossa formed by the lacrimal and maxilla (0) or by the lacrimal and nasal (1) or by maxilla, premaxilla, and lacrimal (2).

27. Pronounced, round accessory antorbital fenestra absent (0) or present (1).

28. Accessory antorbital fossa situated at rostral border of antorbital fossa (0) or situated posterior to rostral border of fossa (1).

237: Dorsal displacement of accessory (maxillary) fenestra: absent (0) or present (1). (modified from Senter *et al.* 2004: char. 5)

239: Accessory antorbital (maxillary) fenestra recessed within a shallow, caudally or caudodorsally open fossa, which is itself located within the maxillary antorbital fossa: absent (0) or present (1).

29. Tertiary antorbital fenestra (fenestra promaxillaris) absent (0) or present (1).

30. Narial region apneumatic or poorly pneumatized (0) or with extensive pneumatic fossae, especially along caudodorsal rim of naris (1).

346. Nasals unfused (0) or fused (1). (Holtz *et al.* 2004)

31. Jugal and postorbital contribute equally to postorbital bar (0) or ascending process of jugal reduced and descending process of postorbital ventrally elongate (1).

246: Jugal does not particulate in margin of antorbital fenestra (0) or participates in antorbital fenestra (1).

32. Jugal tall beneath lower temporal fenestra, twice or more as tall dorsoventrally as it is wide transversely (0) or rod-like (1).

33. Jugal pneumatic recess in caudoventral corner of antorbital fossa present (0) or absent (1).

34. Medial jugal foramen present on medial surface ventral to postorbital bar (0) or absent (1).

35. Quadratojugal without horizontal process caudal to ascending process (reversed ‘L’ shape) (0) or with process (i.e. inverted ‘T’ or ‘Y’ shape) (1).

36. Jugal and quadratojugal separate (0) or quadratojugal and jugal fused and not distinguishable from one another (1).

37. Supraorbital crest on lacrimal in adult individuals absent (0) or dorsal crest above orbit (1) or lateral expansion rostral and dorsal to orbit (2).

38. Enlarged foramen or foramina opening laterally at the angle of the lacrimal above antorbital fenestra absent (0) or present (1).

39. Lacrimal caudodorsal process absent (inverted ‘L’ shaped) (0) or lacrimal ‘T’-shaped in lateral view (1) or rostrodorsal process much longer than posterior process (2).

348. Contact between lacrimal and postorbital: absent (0) or present (1). (Sampson *et al.* 1998)

40. Prefrontal large, dorsal exposure similar to that of lacrimal (0) or greatly reduced in exposure (1) or without exposure (2).

41. Frontals narrow rostrally as a wedge between nasals (0) or end abruptly rostrally, suture with nasal transversely oriented (1).

42. Rostral emargination of supratemporal fossa on frontal straight or slightly curved (0) or strongly sinusoidal and reaching onto postorbital process (1). (Currie, 1995)

43. Frontal postorbital process (dorsal view): smooth transition from orbital margin (0) or sharply demarcated from orbital margin (1). (Currie, 1995).

44. Frontal edge smooth in region of lacrimal suture (0) or edge notched (1). (Currie, 1995)

45. Dorsal surface of parietals flat, lateral ridge borders supratemporal fenestra (0) or parietals dorsally convex with very low sagittal crest along midline (1) or dorsally convex with well developed sagittal crest (2).

46. Parietals separate (0) or fused (1).

219. Supratemporal fenestra bounded laterally and caudally by the squamosal (0) or supratemporal fenestra extended as a fossa on to the dorsal surface of the squamosal (1).

245: Supratemporal fossa with limited extension onto dorsal surfaces of frontal and postorbital (0) or covers most of frontal process of the postorbital and extends rostrally onto dorsal surface of frontal (1). (modified from Currie, 1995)

47. Descending process of squamosal parallels quadrate shaft (0) or nearly perpendicular to quadrate shaft (1).

48. Descending process of squamosal contacts quadratojugal (0) or does not contact quadratojugal (1).

49. Caudolateral shelf on squamosal overhanging quadrate head absent (0) or present (1). (Currie, 1995)

50. Dorsal process of quadrate single headed (0) or with two distinct heads, a lateral one contacting the squamosal and a medial head contacting the braincase (1).

216. Quadrate head covered by squamosal in lateral view (0) or quadrate cotyle of squamosal open laterally exposing quadrate head (1).

51. Quadrate vertical (0) or strongly inclined rostroventrally so that the distal end lies far forward of the proximal end (1).

52. Quadrate solid (0) or hollow, with foramen on caudal surface (1).

53. Lateral border of quadrate shaft straight (0) or with broad, triangular process along lateral edge of shaft contacting squamosal and quadratojugal above an enlarged quadrate foramen (1). (Currie, 1995)

54. Foramen magnum subcircular, slightly wider than tall (0) or oval, taller than wide (1). (Makovicky & Sues 1998)

257. Foramen magnum smaller than or subequal to size of occipital condyle (0) or larger than occipital condyle (1). (Maryánska *et al.* 2002)

55. Occipital condyle without constricted neck (0) or subspherical with constricted neck (1).

56. Paroccipital process elongate and slender, with dorsal and ventral edges nearly parallel (0) or process short, deep with convex distal end (1).

57. Paroccipital process straight, projects laterally or caudolaterally (0) or distal end curves ventrally, pendant (1).

58. Paroccipital process with straight dorsal edge (0) or with dorsal edge twisted rostrolaterally at distal end (1). (Currie, 1995)

59. Ectopterygoid with constricted opening into fossa (0) or with open ventral fossa in the main body of the element (1).

258. Ectopterygoid caudal to palatine (0) or lateral to palatine (1). (Sues, 1997)

259. Caudal limit of vomer terminates rostral to subtemporal fenestra (0) or terminates caudal to suborbital fenestra (1). (modified from Russell & Dong 1993 and Clark *et al.* 2004)

60. Dorsal recess on ectopterygoid absent (0) or present (1).

61. Flange of pterygoid well developed (0) or reduced in size or absent (1).

62. Palatine and ectopterygoid separated by pterygoid (0) or contact (1). (Currie, 1995)

63. Palatine tetraradiate, with jugal process (0) or palatine triradiate, jugal process absent (1). (Elzanowski & Wellnhofer 1996)

64. Suborbital fenestra similar in length to orbit (0) or reduced in size (less than one quarter orbital length) or absent (1). (Clark *et al.* 1994)

## **Mandible**

215. Upper and lower jaws occlude for their full length (0) or diverge rostrally due to kink downward deflection in dentary buccal margin (1).

65. Symphyseal region of dentary broad and straight, paralleling lateral margin (0) or medially recurved slightly (1) or strongly recurved medially (2).

66. Dentary symphyseal region in line with main part of buccal edge (0) or symphyseal end downturned, projects ventral to main body of mandible creating rostral gap (1). (Perez-Moreno *et al.* 1994)

260. Symphyseal region of dentary rostrocaudally narrow, no broader than transverse width of post-symphyseal portion (0) or broader than transverse width of post-symphyseal portion (1). (Clark *et al.* 2004)

261. Axis of dentary symphysis vertical to subvertical (0) or axis of dentary symphysis projects strongly cranially, obliquely oriented with respect to ventral margin of dentary (1).

67. Mandible without coronoid prominence (0) or with coronoid prominence (1).

68. Caudal end of dentary without caudodorsal process dorsal to mandibular fenestra (0) or with dorsal process above rostral end of mandibular fenestra (1) or with elongate dorsal process extending over most of fenestra (2).

69. Labial face of dentary flat (0) or with lateral ridge and inset tooth row (1). (Russell & Dong 1993)

70. Rostral dentary with subparallel dorsal and ventral margins, caudal dentary deepens, minimum dorsoventral height of dentary greater than half the maximum height of dentary (0) or rostral dentary subparallel, caudal dentary deepens, minimum dorsoventral height less than half the maximum height of dentary (1) or entire dentary subtriangular, dentary deepens throughout entire length (2) or dentary extremely rostrocaudally shortened, tapers to a point rostrally (3).

262. Cranioventral margin of dentary straight or dorsally concave (0) or dorsally convex (1). (Rauhut, 2003)

71. Nutrient foramina on external surface of dentary superficial (0) or lie within deep groove (1). (Currie, 1987)

72. External mandibular fenestra oval (0) or subdivided by a spinous rostral process of the surangular (1).

73. Internal mandibular fenestra small and slit-like (0) or large and rounded (1). (Currie, 1995)

74. Foramen in lateral surface of surangular rostral to mandibular articulation, absent (0) or present (1).

75. Splenial not widely exposed on lateral surface of mandible (0) or exposed as a broad triangle between dentary and angular on lateral surface of mandible (1).

250. Splenial forms notched rostral margin of internal mandibular fenestra: absent (0) or present (1). (Currie & Varricchio 2004: char. 35)

76. Coronoid ossification large (0) or only a thin splint (1) or absent (2).

77. Articular without elongate, slender medial, caudomedial, or mediodorsal process from retroarticular process (0) or with process (1).

78. Retroarticular process short, stout (0) or elongate and slender (1).

222. Retroarticular process points caudally (0) or curves gently dorsocaudally (1).

211. Angular exposed almost to end of mandible in lateral view, reaches or almost reaches articular (0) or excluded from posterior end angular suture turns ventrally and meets ventral border of mandible rostral to glenoid (1).

212. Laterally inclined flange along dorsal edge of surangular for articulation with lateral process of lateral quadrate condyle absent (0) or present (1).

79. Mandibular articulation surface as long as distal end of quadrate (0) or twice or more as long as quadrate surface, allowing rostrocaudal movement of mandible (1).

## **Dentition**

345. Teeth in upper jaw homodont (0) or markedly heterodont, rostral teeth incisiform (1).

264. Premaxillary teeth serrated (0) or unserrated (1). (Rauhut, 2003)

80. Premaxilla toothed (0) or premaxilla edentulous (1).

81. Second premaxillary tooth approximately equivalent in size to third and fourth premaxillary teeth (0) or second tooth markedly larger than third and fourth premaxillary teeth (1). (Currie, 1995)

251: First premaxillary tooth size compared with crowns of premaxillary: teeth 2 and 3 slightly smaller or same size (0) or much smaller (1) or much larger (2). (modified from Currie, 1995; Currie & Varricchio 2004: char. 42)

82. Maxillary tooth row extends to level of preorbital bar or slightly rostral to bar (0) or completely antoribital, tooth row ends significantly rostral to the preorbital bar (1) or maxilla edentulous (2). (modified from Osmólska *et al.* 2004; Rauhut, 2003)

248: Maxillary teeth almost perpendicular to jaw margin (0) or inclined strongly caudoventrally (1). (modified from Currie & Varricchio 2004: char. 40)

249: Maxillary tooth height highly variable with gaps evident for replacement (0) or almost isodont with no replacement gaps (1). (Currie & Varricchio 2004: char. 41)

340. Rostral-most maxillary teeth subequal in size (0) or rostral-most maxillary teeth strongly reduced in size compared to adjacent maxillary teeth (1).

83. Maxillary and dentary teeth serrated (0) or some without serrations rostrally (except at base in *S. mongoliensis*) (1) or all without serrations (2).

263. Rostral-most dentary teeth not conical (0) or conical (1).

84. Dentary teeth large, less than 25 in dentary (0) or large number of small teeth (>25) (1) or small number of dentary teeth (<11) (2).

220. Dentary fully toothed (0) or only with teeth rostrally (1) or fully edentulous (2) or rostrally edentulous (3).

85. Dentary teeth in separate alveoli (0) or set in open groove (1). (Currie, 1987)

265. Dentary teeth similar size across tooth row (0) or rostral-most dentary teeth markedly elongate (1). (modified from Russell & Dong 1993)

266. Dentary and maxillary teeth (excluding rostral-most dentary teeth) recurved, subtriangular (ziphodont) (0) or nearly symmetrical in labial view (1). (Xu *et al.* 1999a)

267. Dentary and maxillary teeth (excluding rostral-most dentary teeth) labiolingually compressed, subtriangular, and blade-like (0) or lanceolate with basal cross-section subcircular (1) or conical (2). (modified from Clark *et al.* 1994; state (2) added from Senter, 2007)

86. Serration denticles on dentary and maxillary teeth large (0) or small (1). (Farlow *et al.* 1991)

247: Rostral and caudal denticles of teeth not significantly different in size (0) or rostral denticles, when present, significantly smaller than caudal denticles (1).

87. Serrations on caudal dentary and maxillary teeth simple, denticles convex (0) or distal and often mesial edges of teeth with large, hooked denticles that point toward the tip of the crown (1).

88. Teeth constricted between root and crown (0) or root and crown confluent (1).

89. Dentary teeth evenly spaced (0) or rostral dentary teeth smaller, more numerous, and more closely appressed than those in middle of tooth row (1).

90. Dentaries lack distinct interdental plates (0) or with interdental plates medially between teeth (1).

91. In cross section, premaxillary tooth crowns sub-oval to sub-circular (0) or asymmetrical (D-shaped in cross section) with flat lingual surface (1).

## **Axial Skeleton**

92. Number of cervical vertebrae: ≤10 (0) or 12 or more (1).

93. Axial epipophyses absent or poorly developed, not extending past caudal rim of postzygapophyses (0) or large and caudally directed, extend beyond postzygapophyses (1).

94. Axial neural spine flared transversely (0) or compressed mediolaterally (1).

95. Epipophyses of cervical vertebrae placed distally on postzygapophyses, above postzygapophyseal facets (0) or placed proximally, proximal to postzygapophyseal facets (1).

268. In ventral view, minimum transverse width of cranial cervical centrum less than twice the maximum craniocaudal length of centra (0) or elongate, being approximately two to three times as craniocaudally long as transversely wide (1) or hyperelongate, approximately five times as long as wide (2).

96. Cranial cervical centra level with or shorter than caudal extent of neural arch (0) or centra extending beyond caudal limit of neural arch (1).

97. Carotid process on cervical centra absent (0) or present (1).

98. Cranial cervical centra subcircular or square in cranial view (0) or distinctly wider than high, kidney shaped (1). (Gauthier, 1986)

99. Cervical neural spines craniocaudally long (0) or short and centred on neural arch, giving arch an ‘X’-shape in dorsal view (1). (Makovicky & Sue 1998)

100. Cervical centra with one pair of pneumatic openings (0) or with two pairs of pneumatic openings (1). (Gauthier, 1986)

269. Ventral surface of cervical centra flat or convex (0) or with prominent ventral depression (1).

270. Caudolateral margins of ventral cervical centra undeveloped (0) or with prominent crests (1).

271. Cervical neural spines dorsoventrally tall (subequal to or exceeding dorsoventral height of neural arch from centrum to base of neural spine) (0) or moderately developed, less than height of neural arch (1) or strongly reduced (2).

101. Cervical and cranial trunk vertebrae amphiplatyan or amphicoelus (0) or opisthocoelous (1).

102. Cranial trunk vertebrae without prominent hypapophyses (0) or with large hypapophyses (1). (Gauthier, 1986)

103. Parapophyses of caudal trunk vertebrae flush with neural arch (0) or distinctly projected on pedicels (1). (Norell & Makovicky 1999)

104. Hyposphene-hypantrum articulations in trunk vertebrae absent (0) or present (1).

105. Zygapophyses of trunk vertebrae abutting one another above neural canal, opposite hyposphenes meet to form lamina (0) or placed lateral to neural canal and separated by groove for interspinous ligaments, hyposphenes separated (1) or abutting one another above neural canal, opposite hyposphenes meet ventrally and form a transversely expanded intumescence (2).

106. Cervical vertebrae but not dorsal vertebrae pneumatic (0) or cervical and cranial dorsal vertebrae pneumatic (1) or axial pneumaticity extensive, extends through more than half of dorsal vertebrae (2).

107. Transverse processes of cranial dorsal vertebrae long and thin (0) or short, wide, and only slightly inclined (1).

272. Cranial dorsal vertebrae in lateral view: height of prezygadiapophyseal lamina from base of neural spine less than or subequal to height of cranial centrum (0) or neural arch hypaxially inflated, height of prezygadiapophyseal lamina significantly greater than height of cranial centrum (1).

273. Parapophyseal facets on cranial dorsal vertebrae moderate in size (less than half height of cranial articular facet of centrum) (0) or hypertrophied (greater than 2/3rds of cranial centrum height) (1).

274. Cranial dorsal vertebrae cranial and/or caudal infrazygapophyseal fossae singular (0) or with one or more accessory cranial or caudal centrodiapophyseal laminae dividing the infrazygapophyseal fossae into multiple pneumatic chambers (1).

275. Transverse processes of cranial dorsal vertebrae subhorizontal to inclined (0) or pendant (1).

276. Neural spines of dorsal vertebrae without bifurcation in dorsal view (0) or spine caudally bifurcated, spine subtriangular in dorsal view, caudal margin convex (1) or spine cranially and caudally bifurcated, medially pinched in dorsal view (2).

277. Neural spines on middle dorsal vertebrae dorsoventrally tall (subequal to or greater in height than caudal articular facet of centrum) (0) or short, less than 3/4ths the dorsoventral height of caudal articular facet (1).

108. Neural spines of dorsal vertebrae, in cranial view not transversely expanded distally (0) or expanded to form ‘spine table’ (1).

209. Neural spines on caudal dorsal vertebrae in lateral view rectangular or square (0) or fan-shaped, with craniocaudally expanded dorsal ends (1) or dorsal borders curved, but not craniocaudally expanded (2).

109. Scars for interspinous ligaments terminate at apex of neural spine in dorsal vertebrae (0) or terminate below apex of neural spine (1).

110. Number of sacral vertebrae: 5 (0) or 6 (1) or 7 (2) or 8 (3) or 9 (4).

111. Sacral vertebrae with unfused zygapophyses (0) or with fused zygapophyses forming a sinuous ridge in dorsal view (1).

278. Sacral neural spines discrete (0) or spines fused into continuous spinal ridge (1).

112. Ventral surface of caudal sacral centra gently rounded, convex (0) or ventrally flattened, sometimes with shallow sulcus (1) or centrum strongly constricted transversely, ventral surface keeled (2).

113. Pleurocoels absent on sacral vertebrae (0) or present on cranial sacrals only (1) or present on all sacrals (2).

114. Last sacral centrum with flat caudal articulation surface (0) or convex articulation surface (1).

279. Pleurocoels absent (0) or present (1) on cranial caudal vertebrae. (Xu *et al.*

2002a)

115. Caudal vertebrae with distinct transition point, from shorter centra with long transverse processes proximally to longer centra with small or no transverse processes distally (0) or vertebrae homogeneous in shape, without transition point (1).

116. Transition point in caudal series begins distal to the 10th caudal (0) or between the 7^th^ and 10th caudal vertebra (1) or proximal to the 7^th^ caudal vertebra.

117. Cranial caudal centra tall, oval in cross section (0) or with box-like centra in caudals I-V (1) or cranial caudal centra laterally compressed with ventral keel (2). (modified from Gauthier, 1986)

118. Neural spines of caudal vertebrae simple, undivided (0) or separated into cranial and caudal alae throughout much of caudal sequence (1). (Russell & Dong 1993)

119. Neural spines on distal caudals form a low ridge (0) or spine absent (1) or midline sulcus in centre of neural arch (2). (Russell & Dong 1993)

120. Prezygapophyses of distal caudal vertebrae between 1/3 and whole centrum length (0) or with extremely long extensions of the prezygapophyses (up to 10 vertebral segments long in some taxa) (1) or strongly reduced as in *Archaeopteryx lithographica* (2).

280. Centra of distal caudal vertebrae elongate, craniocaudal length of centrum greater than twice the maximum transverse width (0) or shortened, craniocaudal length of centrum less than twice width (1). (modified from Sues, 1997)

121. More than 40 caudal vertebrae (0) or 25-40 caudal vertebrae (1) or no more than 25 caudal vertebrae (2).

352. Anterior caudal vertebrae, appearance of transverse processes in dorsal view tapered distally (0) or rectangular or slightly expanded distally (1).

122. Proximal end of chevrons of proximal caudals short craniocaudally, shaft cylindrical (0) or proximal end elongate craniocaudally, flattened and plate-like (1).

123. Distal caudal chevrons are simple (0) or cranially bifurcate (1) or bifurcate at both ends (2).

124. Shaft of cervical ribs slender and longer than vertebra to which they articulate (0) or broad and shorter than vertebra (1).

125. Ossified uncinate processes absent (0) or present (1).

126. Ossified ventral (sternal) rib segments absent (0) or present (1).

127. Lateral gastral segment shorter than medial one in each arch (0) or distal segment longer than proximal segment (1).

128. Ossified sternal plates separate in adults (0) or fused (1).

129. Sternum without distinct lateral xiphoid process caudal to costal margin (0) or with lateral xiphoid process (1).

130. Cranial edge of sternum grooved for reception of coracoids (0) or sternum without grooves (1).

131. Articular facet of coracoid on sternum (conditions may be determined by the articular facet on coracoid in taxa without ossified sternum): craniolateral or more lateral than cranial (0) or almost cranial (1). (Xu *et al.* 1999b)

## **Pectoral Girdle**

281. Furcula V-shaped with straight epicleidea (0) or U- shaped with bowed epicleidea (1).

132. Hypocleideum on furcula absent (0) or present (1).

133. Acromion margin of scapula continuous with blade (0) or cranial edge laterally everted (1).

282. Acromion of scapula enlarged, distinctly emarginated from scapular blade to form a craniodorsally extensive blade (0) or acromion poorly developed, does not rise significantly dorsal to longitudinal axis of scapular blade (1).

283. Distal scapular blade expanded (0) or narrows (1). (adopted from Xu *et al.*

2002a)

284. Dorsal margin of scapular blade smooth (0) or dorsal flange present (1). (adapted from Xu *et al.* 2002a)

134. Caudolateral surface of coracoid ventral to glenoid fossa unexpanded (0) or caudolateral edge of coracoid expanded to form triangular subglenoid fossa bounded laterally by enlarged coracoid tuber (1).

135. Scapula and coracoid separate (0) or fused into scapulacoracoid (1).

136. Coracoid in lateral view subcircular, with shallow ventral blade, caudoventral process poorly developed (0) or subquadrangular with extensive ventral blade (1) or shallow ventral blade with elongate caudoventral process (2).

137. Scapula and coracoid form a continuous arc in caudal and cranial views (0) or coracoid inflected medially, scapulocoracoid ‘L’-shaped in lateral view (1).

221. Caudal edge of coracoid not or only shallowly indented below glenoid (0), or caudal edge of coracoid deeply notched just ventral to glenoid, glenoid lip everted (1).

223. Flange on supraglenoid buttress on scapula absent (0) or present (1). (Nicholls & Russell 1985)

138. Articular surface of glenoid fossa restricted from external aspect of scapula and coracoid (0) or with extension onto external surface of scapula (surface opposite costal surface) (1).

139. Scapula longer than humerus (0) or humerus longer than scapula (1).

### Forelimb

285. Internal tuberosity of humerus well developed, extends from caudomedial aspect of humerus and caudally directed (0) or well developed, medially directed (1) or hypertrophied and delineated from either the humeral head by a distinct notch (2) or poorly developed (3) or well developed, extends confluently from medial humerus, caudally directed and rectangular (4). (modified from Russell & Dong 1993; Zanno, 2006)

286. Deltopectoral crest extends for approximately 1/3rd of humeral length (0) or less than 1/4^th^ or humerus (1) or greater than ½ humeral length (2). (modified from Clark *et al.* 2004)

140. Deltopectoral crest large and distinct, proximal end of humerus quadrangular in cranial view (0) or deltopectoral crest less pronounced, forming an arc rather than being quadrangular (1) or deltopectoral crest very weakly developed, proximal end of humerus with rounded edges (2) or deltopectoral crest extremely long and rectangular (3) or proximal end of humerus extremely broad, triangular in cranial view (4).

141. Cranial surface of deltopectoral crest smooth (0) or with distinct muscle scar near lateral edge along distal end of crest for insertion of biceps muscle (1).

287. Trochanter or crest on caudomedial surface of humeral shaft absent (0) or present (1). (adopted from Xu *et al.* 2002a)

288. Cranial tuberosity on cranial surface of distal humerus, proximal to entepicondyle absent (0) or present (1). (adopted from Xu *et al.* 2002a)

289. Transverse axis of distal humerus deflected laterally at least 30º (0) or transverse axes of distal humerus and proximal humerus parallel (1). (Xu *et al.* 2001)

290. Humeral shaft sigmoid, distal humerus deflected cranially (0) or humerus straight shafted (1). (modified from Xu *et al.* 2001)

291. Entepicondyle of humerus spherical (0) or proximodisally elongate and laterally oriented, crest-like in cranial view (1) or poorly developed, absent (2). (modified from Xu *et al.* 2002a)

292. Groove ascending dorsal to entepicondyle on craniolateral margin of humeral shaft absent (0) or present (1).

293. Transverse width of distal humerus moderately expanded (greater than 2x and less than 2.5x) (0) or significantly expanded (greater than 2.7x) (1) or unexpanded (less than 2x the minimum transverse width of humeral shaft) (2).

343. In cranial view, medial aspect of distal humerus unexpanded, entepicondyle situated proximal to ulnar condyle (0) or medial aspect expanded and subtriangular in cranial view, entepicondyle located well medial to ulnar condyle (1).

294. Ulnar shaft straight or slightly sigmoid (0) or caudally convex (bowed) (1). (modified from Gauthier, 1986)

236. Ulna/femoral length ratio: significantly less than one (0) or equal or greater than one (1).

142. Olecranon process weakly developed (0) or distinct and large (1) or hypertrophied (2).

143. Distal articular surface of ulna flat (0) or convex, semilunate surface (1).

144. Proximal surface of ulna a single continuous articular facet (0) or divided into two distinct fossae (one convex, the other concave) separated by a median ridge (1).

214. Radius and ulna well separated (0) or with distinct adherence or syndesmosis distally (1).

145. Lateral proximal carpal (ulnare?) quadrangular (0) or triangular in proximal view (1).

146. Two distal carpals in contact with metacarpals, covering the base of metacarpal I (and perhaps contacting metacarpal II) covering the base of metacarpal II (0) or a single distal carpal capping metacarpals I and II (1).

147. Distal carpals not fused to metacarpals (0) or fused to metacarpals, forming carpometacarpus (1).

148. ‘Semilunate’ carpal: comprised of distal carpal one only (0) or comprised of distal carpals one and two (1) or absent, distal carpals flattened (2).

213. Distal articular ends of metacarpals I + II ginglymoid (0) or rounded, smooth (1).

149. Metacarpal I half or less than half the length of metacarpal II, and longer proximodistally than wide transversely (0) or subequal in length to metacarpal II (1) or very short and wider transversely than long proximodistally (2).

295. Ventrolateral aspect of proximal surface of metacarpal I, rectangular buttress underlies ventromedial surface of metacarpal II: absent (0) or present (1). (Russell & Dong 1993)

210. Shaft diameter of phalanx I-1 less (0) or greater (1) than shaft diameter of radius.

150. Third manual digit present, phalanges present (0) or reduced to no more than metacarpal splint (1).

296. Metacarpal III *in dorsal view* distally not ginglymoid (0) or ginglymoid (1). (modified from Senter, 2007)

297. Metacarpal II longer than metacarpal III (0) or subequal to metacarpal III (1). (Russell & Dong 1993)

298. Manual phalanx II-I shorter than I-I (0) or longer (1). (Senter, 2007)

299. Metacarpal III straight to slightly sigmoid (0) or laterally convex (1). (Gauthier, 1986)

300. Ratio of metacarpal II(phalanx II-I + phalanx II-II) less than or equal to 100% (0) or greater than 100% (1). (Zanno, 2006)

301. Length of manual PIII-III/sum of lengths of manual PIII-I and manual PIII-II greater than 100% (0) or less than 100% (1). (Gauthier, 1986)

302. Ligament pits on manual phalanges strongly developed (0) or weakly developed or absent (1). (Russell & Dong 1993)

303. Manual unguals subequal in length with penultimate phalanx or shorter (0) or elongate, up to twice as long as penultimate phalanx (1) or lengthening extreme, more than three times as long as penultimate phalanx (2). (modified from Zanno, 2006)

151. All manual unguals strongly curved (0) or weakly curved (1) or nearly straight (2).

152. Unguals on all digits generally similar in size or PIII-IV slightly smaller (0) or digit I bearing large ungual and unguals of other digits distinctly smaller (1) or digit II bearing largest ungual (2).

153. Proximodorsal ‘lip’ on manual unguals - a transverse ridge immediately dorsal to the articulating surface - absent (0) or present only on unguals II and III (1) or present on all manual unguals (2). (Currie & Russell 1988)

349. Dorsal surface of distal portion of manual ungual III-3 rounded (0) or flattened (1).

353. Flexor tubercles of manual unguals large (0) or weak (1).

354. Flexor tubercles of mannual unguals positioned ventral to articular facet (0) or displaced distally from articular end (1).

355. Ventral process ventral to the articular surface of manual unguals absent (0) or present (1).

356. Lateral and medial grooves of manual unguals extends to proximal end (0) ends more distally (1).

## **Pelvic Girdle**

154. Ventral edge of cranial ala of ilium straight or gently curved (0) or ventral edge with shallow, obtuse process (1) or process strongly hooked (2).

155. Preacetabular part of ilium roughly as long as postacetabular part of ilium (0) or more than 1.5 times length of postacetabular part of ilium (1). (Clark *et al.* 2004)

156. Cranial end of ilium gently rounded or straight (0) or cranial end strongly convex, lobate (1) or pointed at craniodorsal corner with concave cranioventral edge (2).

304. Ventral blade of preacetabular portion of ilium parasagital, inline with dorsal portion (0) or gently deflected laterally (1) or strongly deflected laterally, nearly perpendicular to parasagittal plane of ilium. (modified from Kirkland *et al.* 2005)

305. In lateral view, preacetabular portion of ilium (including ventral process) poorly developed, subequal with height of the ilium directly dorsal to the centre of the acetabulum (0) or hyperelongate, at least twice the height (1).

157. Supraacetabular crest on ilium as a separate process from antitrochanter, forms ‘hood’ over femoral head present (0) reduced, not forming hood (1) or absent (2).

158. Postacetabular ala of ilium in lateral view squared (0) or acuminate (1) or reduced, iliac blade terminates at rectangular end just posterior to level of acetabulum (2).

226. Dorsal edge of postacetabular blade dorsally convex or straight (0) or dorsally concave brevis shelf does not contribute to concavity, postacetabular process squared (1) or dorsally concave, brevis shelf extending caudal to vertical face of ilium giving ilium a dorsally concave outline in lateral view, postacetabular process rounded or acuminate and severely reduced (2). (Novas, 2004)

306. Dorsal margins of medial ilium closely appressed, nearly contacting sacral neural spines in dorsal view (0) or broadly separated (1). (adapted from Sues, 1997)

307. Angle between dorsal margin of ilium and line connecting articular facets for pubis and ischium less than 15 degrees (gently inclined) (0) or between 15 and 35 degrees (moderately inclined) (1) or more than 35 degrees (steeply inclined).

227. Postacetabular end of ilium terminating in rounded or square end in dorsal view (0) or with lobate brevis shelf projecting from end of ilium and beyond end of postacetabular lamina (1).

159. Postacetabular blades of ilia in dorsal view subparallel (0) or diverge caudally (1).

160. Tuber along dorsal edge of ilium, dorsal or slightly caudal to acetabulum absent (0) or present (1).

308. Dorsal surface of postacetabular portion of ilium in dorsal view smooth, nonrugose (0) or with rugosity causing transverse expansion of the caudal dorsal margin (1) or hyperrugose, with hypertrophied caudal tuberosity (2).

161. Brevis fossa shelf-like (0) or deeply concave with lateral overhang (1) or strongly reduced, nearly absent (2).

217. Brevis fossa poorly developed adjacent to ischial peduncle and without lateral overhang, medial edge of brevis fossa visible in lateral view (0), or fossa well developed along full length of postacetabular blade, lateral overhang extends along full length of fossa, medial edge completely covered in lateral view (1).

162. Antitrochanter caudal to acetabulum absent or poorly developed (0) or prominent (1).

163. Ridge bounding cuppedicus fossa terminates rostral to acetabulum or curves ventrally onto cranial end of pubic peduncle (0), or rim extends far caudally and is confluent or almost confluent with acetabular rim (1).

164. Cuppedicus fossa deep, ventrally concave (0) or fossa shallow or flat, with little or no lateral overhang (1) or absent (2).

309. Pubic peduncle of ilium craniocaudally elongate and narrow, craniocaudal depth to transverse width ratio of pubic peduncle of ilium approximately 2 (0) or equidimensional and roughly triangular in outline (1) severely compressed craniocaudally, transverse dimension measuring more than twice the craniocaudal depth (2). (modified Xu *et al.* 1999a; Rauhut, 2003)

310. Pubic peduncle of ilium straight (0) or anterior margin straight, posterior margin curved, articular surface ventrally directed (1) or anterior margin straight, posterior margin curved, articular surface caudoventrally directed (2) or anterior and posterior margins both curved, articular surface caudoventrally directed (3).

311. Pubic peduncle of ilium approximately twice the length of the ischiadic peduncle (0) or subequal in length with ischiadic peduncle (1) or pubic peduncle hyperelongate, almost 3x the length of the ischiadic peduncle (2) or ischiadic peduncle significantly longer than pubic peduncle (3). (modified from Gauthier, 1986; Rauhut, 2003; Senter, 2007)

312. Ischiadic peduncle of ilium and antitrochanter (if present) divisible (0) or functional division between antitrochanter and ischiadic peduncle indistinguishable, both structures form enlarged ventrolaterally flattened boss (1) or boss hypertrophied and spherical (2).

313. Contact between ischium and ilium flat or slightly concave (0) or iliac peduncle of ischium with deep cavity for insertion of peg-shaped, ventrally tapering ischiadic peduncle of ilium (1). (Kirkland *et al.* 2005)

314. Pubic peduncle of ischium does not extend beyond ventral margin of ischiadic peduncle of pubis (0) or pubic peduncle with pronounced ventral process and well developed notch (1).

165. Caudal edge of ischium straight (0) or with proximal medial caudal process (1).

232. Ventral caudal process of ischium absent (0) or present (1). (modified Ostrom, 1976; Forster *et al.* 1998)

315. Ventral caudal process of ischium located entirely proximal to the obturator process (0) or located opposite obturator process (1) or process extends distal to obturator process (2).

230. Proximodorsal process of ischium small, tab-like or pointed process along caudal edge of ischium (0) or process large proximodorsally hooked and separated from iliac peduncle of the ischium by a notch (1). (Chiappe *et al.* 1999)

166. Ishium with rodlike shaft (0) or with wide, flat, and plate-like shaft (1). (modified from Forster *et al.* 1998; Xu *et al.* 1999b)

167. Ischiadic shaft straight (0) or ventrodistally curved cranially (1) or hooked caudally (2).

168. Lateral face of ischiadic blade flat [or round in rodlike ischia] (0) or laterally concave (1) or with longitudinal ridge subdividing lateral surface into cranial (including obturator process) and caudal parts (2).

169. Obturator process of ischium absent (0) or proximal in position (1) or located near middle of ischiadic shaft (2) or located at distal end of ischium (3).

233. Obturator process square (i.e. with distinct caudal edge or notch) (0) or triangular with caudal end confluent with shaft (1). (modified from Forster *et al.* 1998)

234. Triangular obturator process with short rostral projection and wide base along ischial shaft (0) or with short base, long process extending rostrally (1).

170. Obturator process does not contact pubis (0) or contact with pubis incomplete, restricted to dorsal aspect (1) or contact expansive, obturator process contacts pubis over entire dorsoventral length (2).

255. Obturator process does not contact pubis (0) or contacts pubis, process equidimentional (1) or process elongate, twice as craniocaudally long as dorsoventrally tall (2).

171. Obturator notch on pubis well developed with significant ventral extension of ischiadic peduncle (0) or slight, poorly developed (1) or notch or foramen absent (2).

172. Semicircular scar on caudal part of the proximal end of the ischium, absent (0) or present (1).

173. Ischium more than two-thirds (0) or two-thirds or less of pubis length (1).

174. Distal ends of ischia form symphysis (0) or approach one another but do not form symphysis (1) or widely separated (2).

175. Ischial boot (expanded distal end) present (0) or absent (1).

176. Tubercle on cranial edge of ischium absent (0) or present (1)*.*

316. Contact between ischium and pubis flat (0) or markedly sinuous (1).

317. Pubic shaft rodlike (0) or mediolaterally flattened (1).

177. Pubis propubic (0) or pubis vertical (1) or pubis caudally oriented (opisthopubic) (2).

178. Pubic boot absent, no craniocaudal projections on distal pubis (0) pronounced, projects cranially and caudally (1) caudally focused, with little or no cranial process (2) or cranially focused, little to no caudal process (3).

179. Shelf on pubic shaft proximal to symphysis (‘pubic apron’) extends medially from middle of cylindrical pubic shaft (0) or shelf extends medially from cranial edge of craniocaudally flattened shaft (1) or extends caudally from caudal to caudomedial aspect of pubic shaft (2).

180. Pubic shaft straight (0) or distal end curves cranially, cranial surface of shaft concave (1) or shaft curves caudally, cranially convex curvature (2). See also (Calvo *et al.* 2004)

231. Lateral face of pubic shaft smooth (0) or with prominent lateral tubercle about halfway down the shaft (1). (Senter *et al.* 2004)

181. Pubic apron about half of pubic shaft length (0) or less than 1/3 of shaft length (1).

182. Contact between pubic apron contributions of both pubes meet extensively (0) contact disrupted by a slit (1) or no contact (2).

318. Distal pubic shaft subequal in craniocaudal depth with proximal shaft or thinner (0) or greatly enlarged, more than twice the craniocaudal depth of the proximal shaft (1).

### Hind limb

319. Femur/tibia ratio less than 100% (0) or greater than 100% (1). (modified from Xu *et al.* 1999a)

183. Femoral head without fovea capitalis (for attachment of capital ligament) (0) or circular fovea present in centre of medial surface of head (1).

320. Femoral head extends perpendicular to long axis of femur or with slight ventral decline (0) or femoral head dorsally inclined, extending at an obtuse angle from long axis of femur (1).

321. Region bridging femoral head and greater trochanter confluent in craniocaudal width in dorsal view (0) or region constricted in dorsal view (1).

339. Greater trochanter and femoral head confluent in cranial view (0) or bridging region depressed (1).

322. Greater trochanter and femoral head subequal in craniocaudal depth or greater trochanter smaller (0) or greater trochanter significantly expanded in craniocaudal depth relative to femoral head (1). (modified from Rauhut, 2003)

323. Femoral head demarcated from neck by a raised ventral rim (0) or femoral head confluent with neck, no noticeable delineation (1).

324. Ligament groove on caudal surface of femoral head or moderate to deeply formed, bounded medially by a well developed caudal lip (0) or absent to poorly developed and shallow (1). (Rauhut, 2003)

325. Lesser trochanter terminates ventral to the level of the greater trochanter in cranial view (0) or lesser trochanter terminates subhorizontally with greater trochanter (1) or fused with greater trochanter (2). (modified from Makovicky & Sues 1998, state (2) new)

184. Lesser trochanter separated from greater trochanter by deep cleft (0) or trochanters separated by small groove (1) or completely fused (or absent) to form a trochanteric crest (2).

185. Lesser trochanter of femur alariform (0) or cylindrical in cross section (1).

218. Vertical ridge on lesser trochanter present (0) or absent (1).

186. Lateral ridge absent or represented only by faint rugosity (0) or distinctly raised from shaft, mound-like (1).

187. Fourth trochanter on femur present (0) or absent (1).

188. Accessory trochanteric crest distal to lesser trochanter absent (0) or present (1).

189. Cranial surface of femur proximal to medial distal condyle without longitudinal crest (0) or crest present extending proximally from medial condyle on cranial surface of shaft (1).

190. Popliteal fossa between end of femur open distally (0) or closed off distally by contact between distal condyles (1).

350. Femur in lateral view curved (0) or nearly straight (1).

351. Lateral distal condyle of the femur extends farther distally than the medial condyle (0) or extends about as far distally as the medial condyle (1).

191. Fibula reaches proximal tarsals (0) or short, tapering distally, and not in contact with proximal tarsals (1).

192. Medial surface of proximal end of fibula concave along long axis (0) or flat (1).

193. Deep oval fossa on medial surface of fibula near proximal end absent (0) or present (1).

194. Distal end of astragalus and calcaneum with condyles separated by shallow, indefinite sulcus (0) or with distinct condyles separated by prominent tendinal groove on cranial surface (1).

195. Medial cnemial crest absent (0) or present on proximal end of tibia (1).

326. Lateral fossa of proximal tibia (incisura tibialis) deeply notched (0) or wide and shallow, nearly absent (1).

327. Craniocaudal length of proximal tibia exceeds transverse width (0) or proximal tibia transversely wider (1).

328. Fibular crest on tibia proximally positioned (0) or long extends to midshaft (1).

341. Fibular crest on tibia short less than 1/5^th^ the length of tibia (0) or long (1/4 to 1/3^rd^ the length of tibia) (1).

342. Mediocranial aspect of distal tibia covered by astragalus (0) or mediocranial apect of distal tibia exposed, yet unmodified (1) or craniomedial distal tibia exposed, developed into a cranial tuberosity (2).

329. Proximal fibular margin subhorizontal (0) or cranial portion dorsally elevated (1). (Xu *et al.* 2002a)

330. Trochanter for insertion of m. iliofibularis on fibular shaft proximally positioned (0) or positioned at midshaft (1). (Xu *et al.* 2002a)

331. Cranial and caudal aspect of proximal fibula subequal in transverse width (0) or transverse width of proximal fibula narrows caudally (1). (modified from Xu *et al.* 2002a)

196. Ascending process of the astragalus tall and broad, covering most of cranial surface of distal end of tibia (0) or process short and slender, covering only lateral half of cranial surface of tibia (1) or ascending process tall, but with medial notch that restricts it to lateral side of cranial face of distal tibia (2) or process extends lateral to the tibial shaft, contacting fibula (3). (state (3) adopted from Russell & Dong 1993)

197. Ascending process of astragalus confluent with condylar portion (0) or separated by transverse groove or fossa across base (1).

332. Lateral condyle of astragalus well developed (0) or strongly reduced, lateral tibia exposed on distal and cranial surfaces (1). (modified from Paul, 1984; Clark *et al.* 2004)

198. Astragalus and calcaneum separate from tibia (0) or fused to each other and to the tibia in late ontogeny (1).

199. Distal tarsals separate, not fused to metatarsals (0) or form metatarsal cap with intercondylar prominence that fuses to metatarsal early in postnatal ontogeny (1).

333. Metatarsus elongate, metatarsals II-IV much longer than wide (0) or short and broad, metatarsals II-IV less than twice as long as they are together broad (1). (Clark *et al.* 2004)

334. Metatarsus more than 45% the length of the tibia (0) or between 40-38% (1) or less than 36% (2). (Russell & Dong 1993; Xu *et al.* 1999a)

335. Shafts of metatarsals not appressed (0) or appressed throughout entire metatarsus (1). (Senter, 2007)

200. Metatarsals not co-ossified (0) or co-ossification of metatarsals begins proximally (1) or distally (2).

201. Distal end of metatarsal II smooth, not ginglymoid (0) or with developed ginglymus (1).

235. Tuber along extensor surface of MT II absent (0) or present (1). (Chiappe, 2002).

202. Distal end of metatarsal III smooth, not ginglymoid (0) or with developed ginglymus (1).

203. MT III proximal shaft prominently exposed between MT II and MT IV along entire metapodium (0) or MT III proximal shaft constricted and much narrower than either II or IV, but still exposed along most of metapodium, subarctometatarsal (1) or very pinched, not exposed along proximal section of metapodium, arctometatarsal (2) or proximal part of MT III lost (3). (Novas & Pol 2005:char. 200)

229. Large, longitudinal flange along caudal or lateral face of metatarsal IV absent (0) or present (1). (modified from Novas & Pol 2005)

204. Ungual and penultimate phalanx of pedal digit II similar to those of III (0) or penultimate phalanx highly modified for extreme hyper-extension, ungual more strongly curved and significantly larger than that of digit III (1).

228. Flexor heel on phalanx II-2 small and asymmetrically developed only on medial side of vertical ridge subdividing proximal articulation (0) or heel long and lobate, with extension of midline ridge extending onto its dorsal surface (1).

205. Metatarsal I articulates with the middle of the medial surface of metatarsal II (0) or metatarsal I attaches to caudal surface of distal quarter of metatarsal II (1) or metatarsal I articulates to medial surface of metatarsal II near its proximal end (2) or metatarsal I absent (3).

206. Metatarsal I attenuates proximally, without proximal articulating surface (0) or proximal end of metatarsal I similar to that of metatarsals II-IV (1).

207. Shaft of MT IV round or thicker dorsoventrally than wide in cross section (0) or shaft of MT IV mediolaterally widened and flat in cross section (1).

336. Proximal end of metatarsal IV curls around plantar side of proximal end of metacarpal III (0) or does not (1). (Senter, 2007)

208. Foot symmetrical (0) or asymmetrical with slender MTII and very robust MT IV, excluding flange (1).

337. Pedal unguals III and IV straight or weakly curved (0) or strongly curved (1). (Senter, 2007)

338. Ungual of pedal digits III-IV shorter in length to or slightly longer than penultimate phalanx (0) or twice as long or more than the penultimate phalanx (1). (Clark *et al.* 2004)
